# Supplementary material for: Developing and validating a school-based screening tool of Fundamental Movement Skills (FUNMOVES) using Rasch analysis
Source: PLoS One. 2021 Apr 16;16(4):e0250002. doi: 10.1371/journal.pone.0250002 (PMC8051776; doi:10.1371/journal.pone.0250002)
Supplement: S1 File — (DOCX) [file pone.0250002.s001.docx]

**S1 File. Teacher Manual from study 3.**


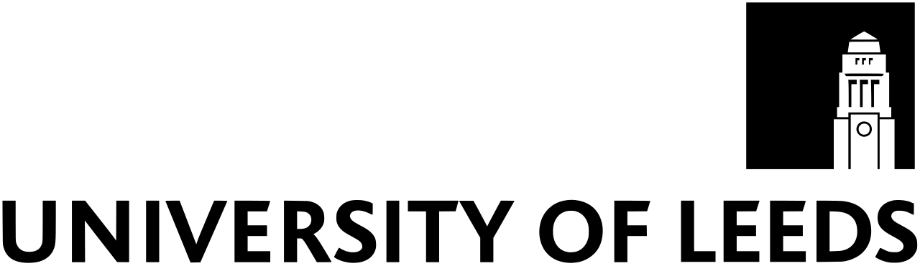

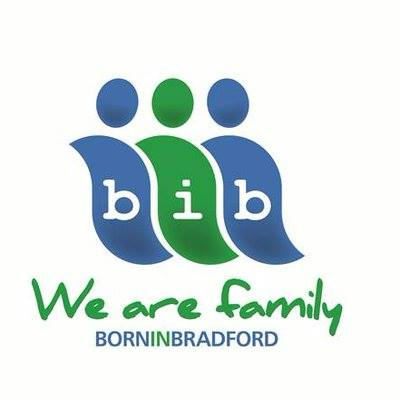


Lucy Eddy [ps13lhe]

University of Leeds | L.Eddy@leeds.ac.uk

FUNMOVES manual

A User guide for setting up, implementing and scoring the activities

About FUNMOVES

FUNMOVES is a tool designed to **assess** **fundamental movement skills** in children. It specifically focuses on a number of motor skills that are central to childhood development: running, jumping, hopping, throwing, kicking and balancing.

**Why are fundamental movement skills important?**

Research has shown that children who have **poor fundamental movement skills** have an **increased risk** of adverse outcomes in childhood including **physical and mental health problems**, as well as **poor academic achievement**. Identifying children who struggle with key motor skills will help schools to target support effectively for those pupils.

**Why should I use FUNMOVES?**

FUNMOVES is an **evidence-based** assessment tool which has been **modified** based on the **teacher feedback** to ensure that it is feasible for use in schools. FUNMOVES is a fast way to identify children in your school which may need additional support – it can assess a whole class in two PE lessons. After completing the assessment, you will receive **tailored reports** on how each child performed compared to other children in the same year group.

Preparing for FUNMOVES

**Resources Required**

- 25 beanbags
- 80m electrical tape / chalk
- 6 response sheets
- 5 team score sheets
- A measuring tape or meter ruler
- A stopwatch or a device able to time activities
- Pens
- The help of a second member of staff to score activities

**Splitting your class into teams**

1. **Separate your class into groups of five, based on their ability**
   When you are splitting them into groups you should consider how good each child is at running, jumping, hopping, throwing, kicking and balancing.
2. **Complete the demographic information on the response sheet**Dominant hand should be noted as the hand they write with. You should state that you think a child has a motor problem if they have difficulty with handwriting, are clumsy when moving around the classroom, or has difficulty physically interacting with objects.


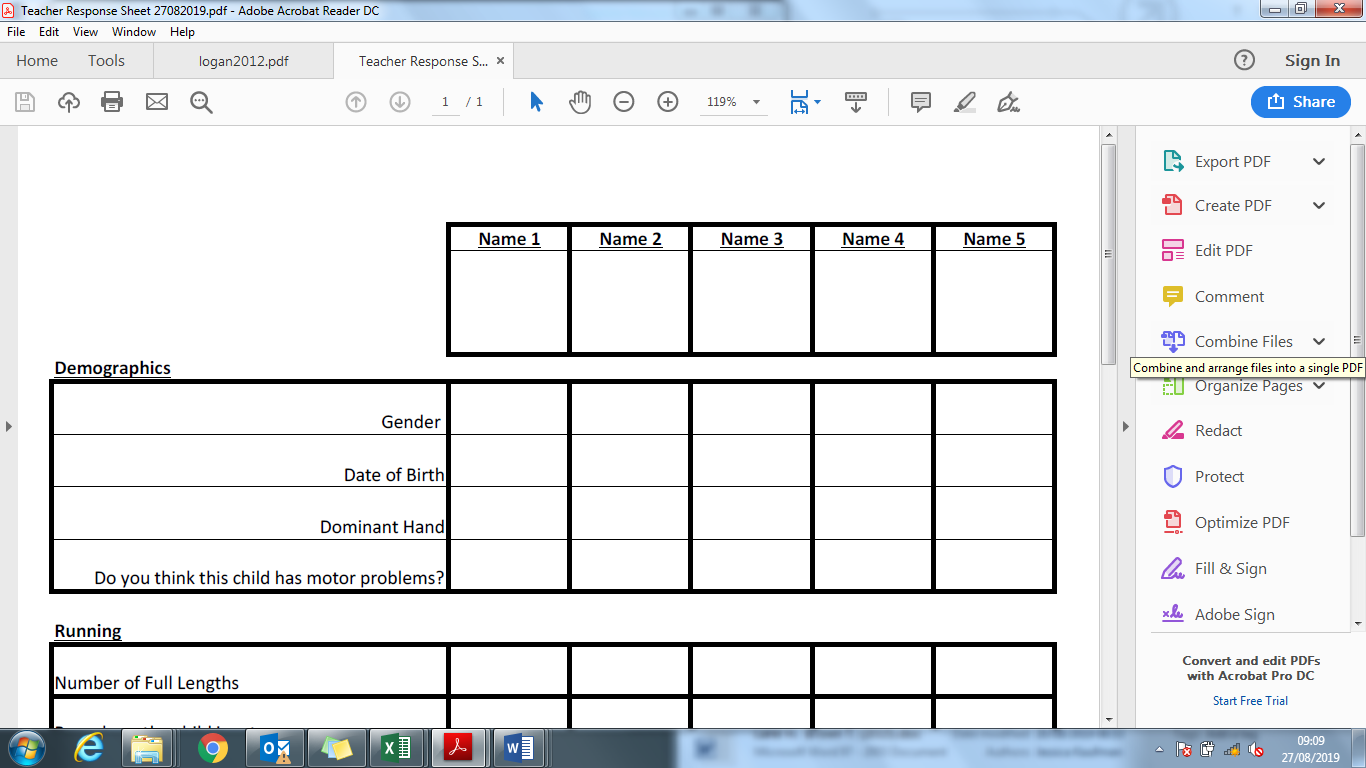


1. **Compile teams**All the children whose names are written under name 1 will be a team, all the children who are in the name 2 column make up a team etc.
2. **Choosing team names**Once the teams are established, you should give each team a team score sheet, on which they can write their names and decide upon a team name.

**Setting up the grid**


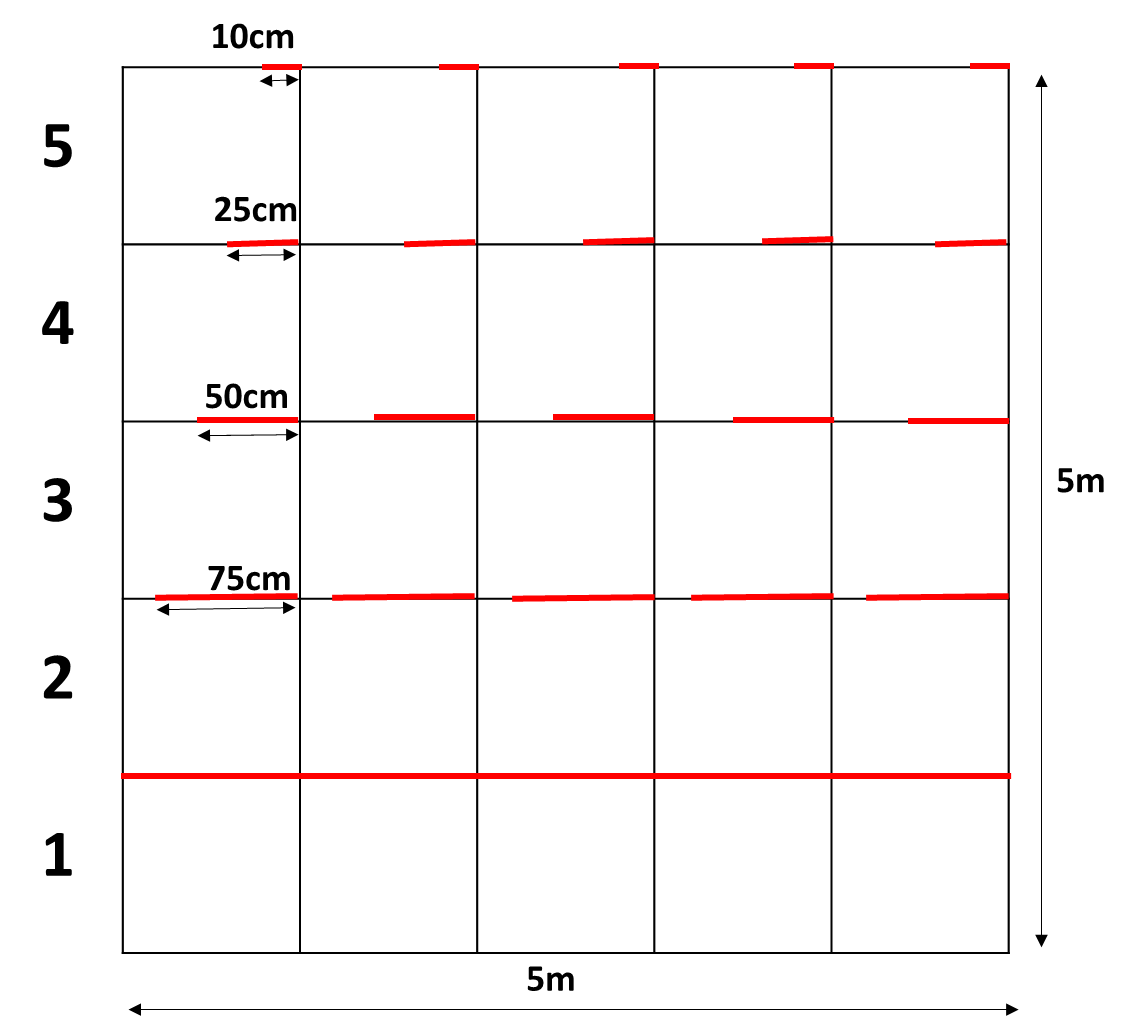
All activities are based within a 5x5 metre grid, which should be set up using electrical tape or chalk and a tape measure to the specifications shown below. Please note the red lines on the diagram should be marked out in a different colour to the rest of the grid. Make sure there is space at the bottom of the grid for your class to sit in their teams.

Implementing FUNMOVES

**Team Competition**

FUNMOVES can be run as a **team competition** to make it fun and engaging. A class can complete the assessment in approximately **1 hour**, and can be split up over a number of **PE lessons**.

- Give each team a score sheet and a pen so they can count up the number of points they get
- Before you begin testing, line up the children in their teams, in the box on your response sheet which corresponds to the lane on the grid
- Go through the activities one at a time, testing all children before moving to the next activity
- Do not allow children to practice the tasks before testing
- To avoid children not completing the tasks properly, tell them that they will not receive any points for their team if they cheat
- Try not to make it obvious when a child has been unsuccessful at completing a task, or if a child is ‘winning’
- Mark down unsuccessful attempts on the response sheet, but allow the children to carry on and complete all activities, regardless of the level they achieved


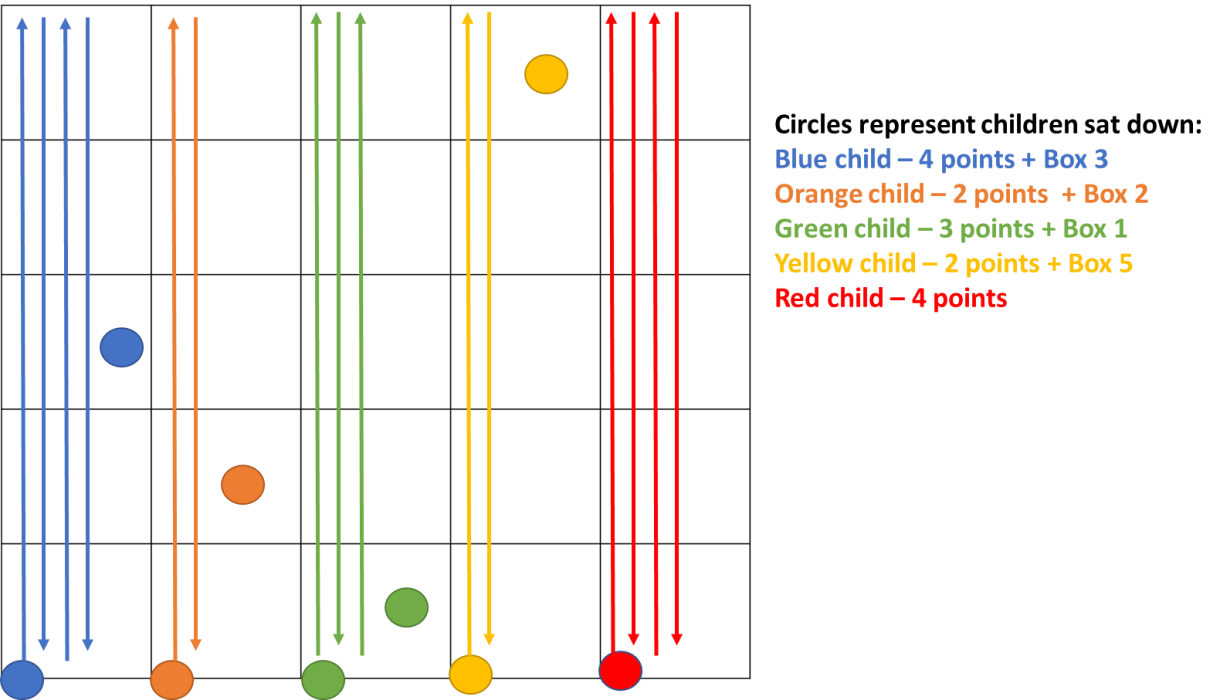
Running

**Rules**

- Children have **15 seconds** to run as many times from the start line to the far line and back as possible
- When you say **STOP**, they have to **sit down** as quickly as possible facing the way they were running
- **Foot** must **touch** both the start **line** and the far line of the grid for them to get any points

**Demonstrate the activity and explain the rules**

**Scoring**


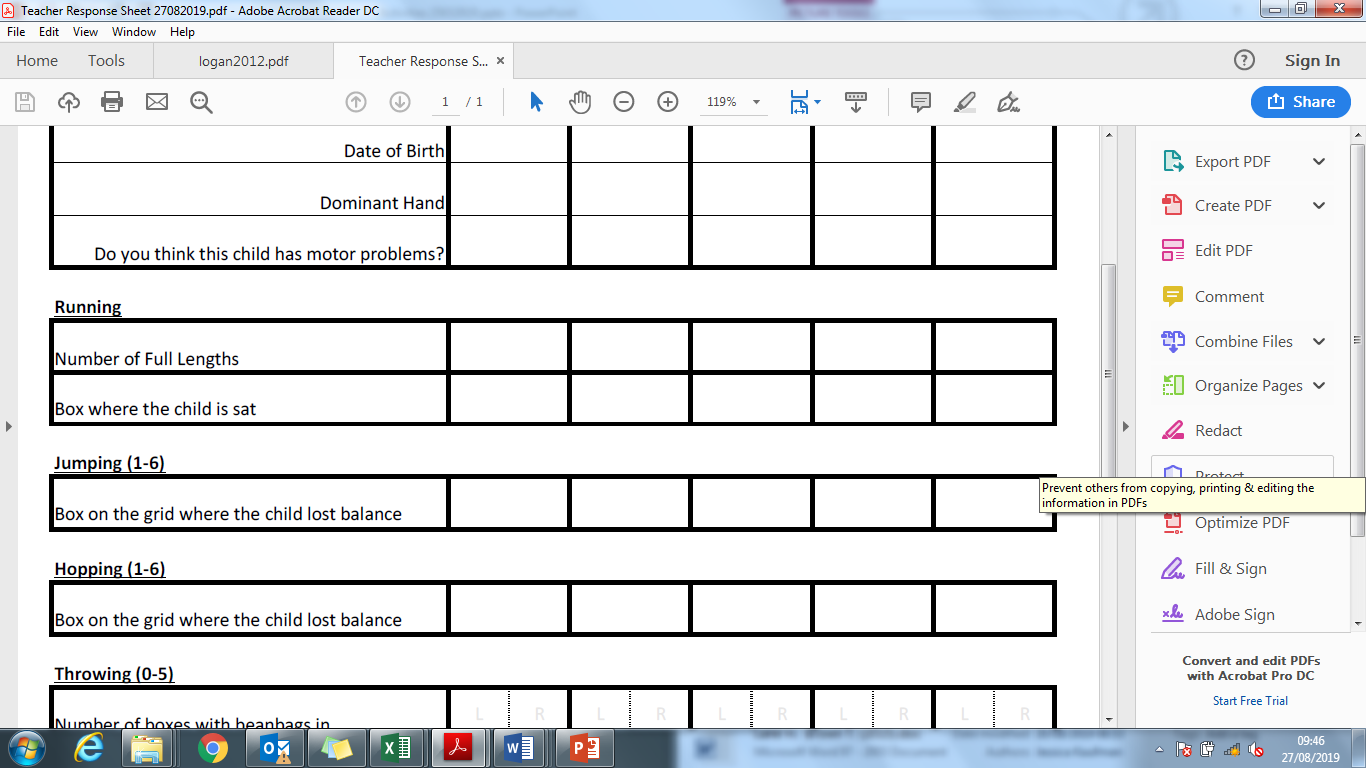
Children are scored by the number of ‘**full lengths**’ they have run

- A **full length** comprises a **5 metre** run (from one side of the grid to the other)


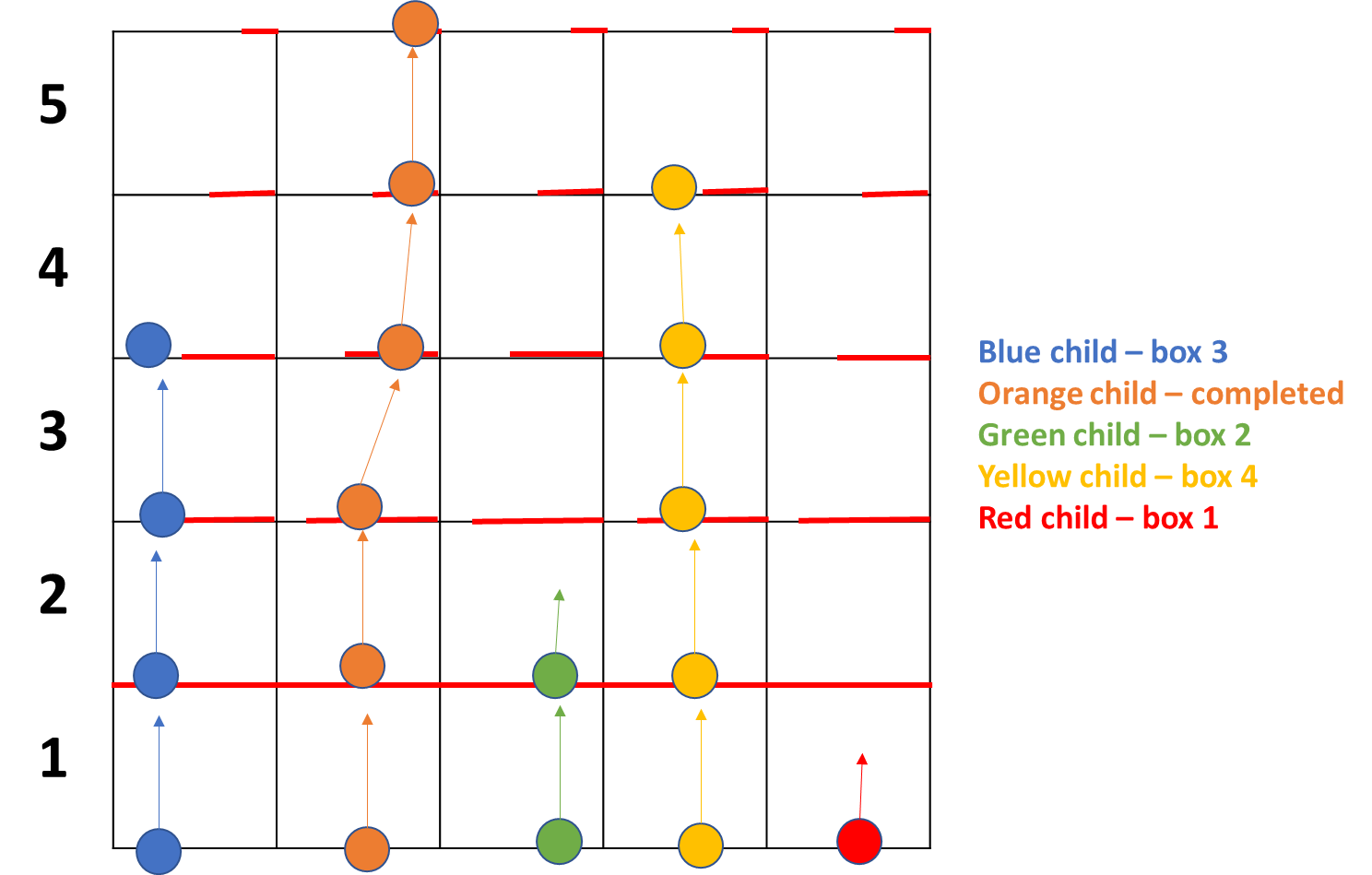
Jumping

**Rules**

- Children **jump** to the first line, then pause
- Children are not allowed to jump line to line, they must use **small jumps**
- When all five children reach the line, count **3 seconds**, and then set them off to the next line
- Children must land with both feet in the red zone each time

**Demonstrate the task and explain the rules**

**Scoring**


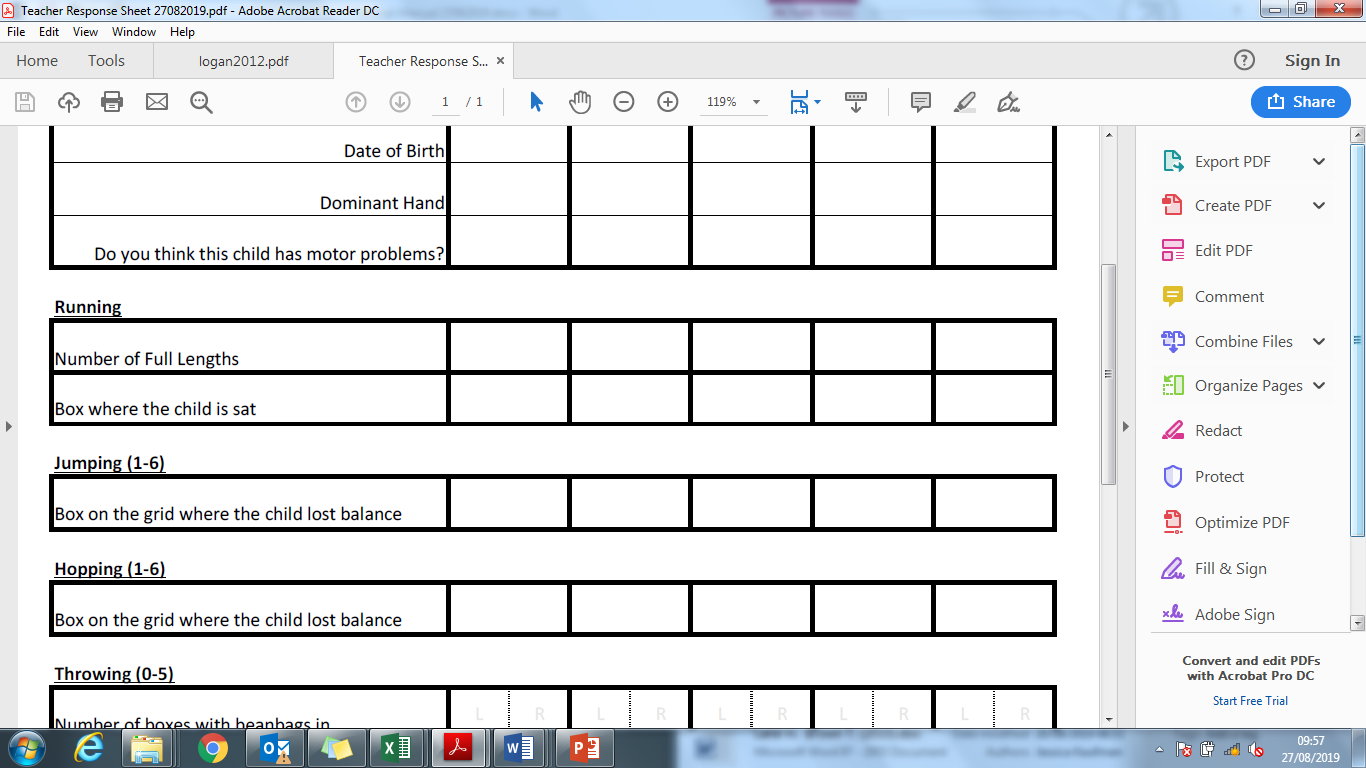
Children are scored by the **box** in which they lost **balance**.

- Losing balance includes
  - Falling
  - Pausing not on the line
  - One or both of the child’s feet is not in the red zone
- If the child loses balance on the line, mark that they lost balance in the box before
   e.g. if the child lost balance on the line between boxes 2 and 3, put a cross in box 2
- If a child completes the task, give them a score of 6


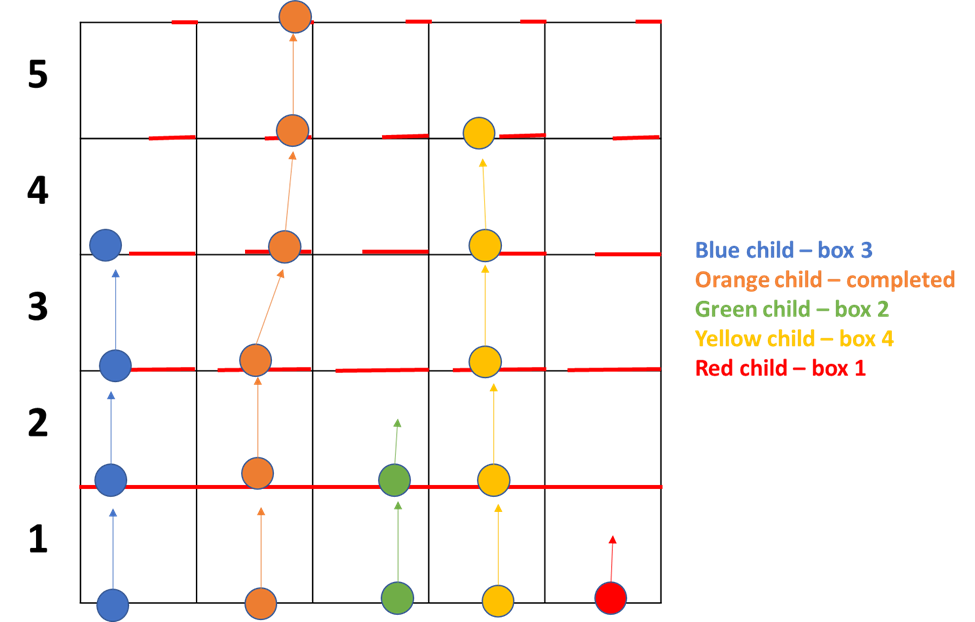
Hopping

**Rules**

- Children **hop on one leg** to the first line, then pause (whichever leg they want)
- Children are not allowed to hop line to line, they must use **small hops**
- When all five children reach the line, count **3 seconds**, and then set them off to the next line
- Children must not put their foot down at any point during the activity
- Children cannot change the leg on which they hop during the activity
- Children must land in the red zone on each line

  **Demonstrate the task and explain the rules** **Scoring**


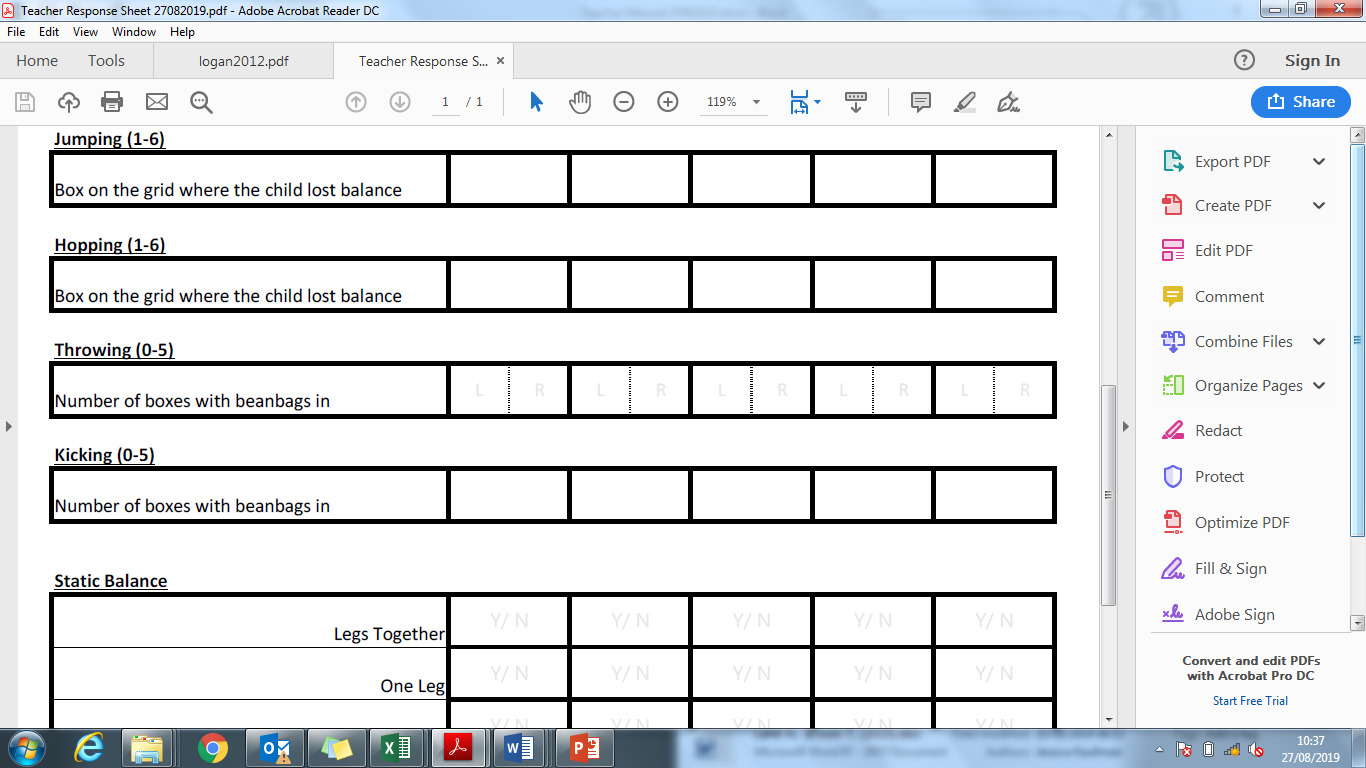
Children are scored by the **box** in which they lost **balance.**

- Losing balance includes
  - Falling
  - Putting their foot down
  - Pausing not on the line
  - Foot shuffling whilst pausing on the line
  - Not landing within the red zone on the line
- If the child loses balance on the line, mark that they lost balance in the box before
   e.g. if the child lost balance on the line between boxes 2 and 3, put a cross in box 2
- If a child completes the task, give them a score of 6

Throwing

**Set up**

- Each child needs 5 beanbags that are all the same colour
-
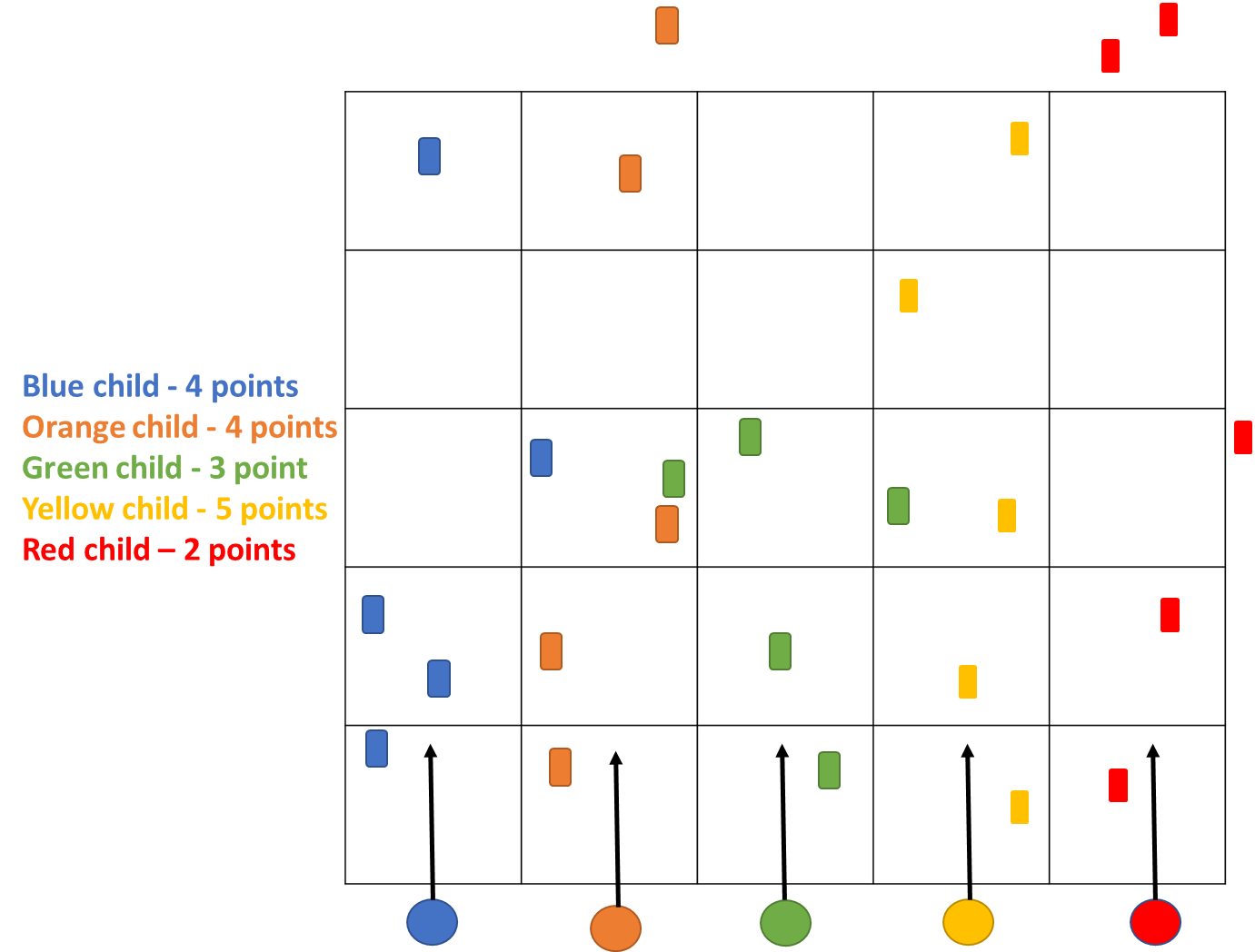
Children stood next to one another on the grid should not have the same colour beanbags

**Rules**

- Children aim to throw (underarm) one beanbag into each box in their lane
- Foot needs to be behind the line
- Each child can only throw one beanbag at a time
- Points are only be awarded for beanbags that land in their lane
- Only one point can be awarded per box in their lane
- Get all children to do the task right handed (all 5 beanbags), then reset the task and allow them to complete it left handed

**Demonstrate the task and explain the rules**

**
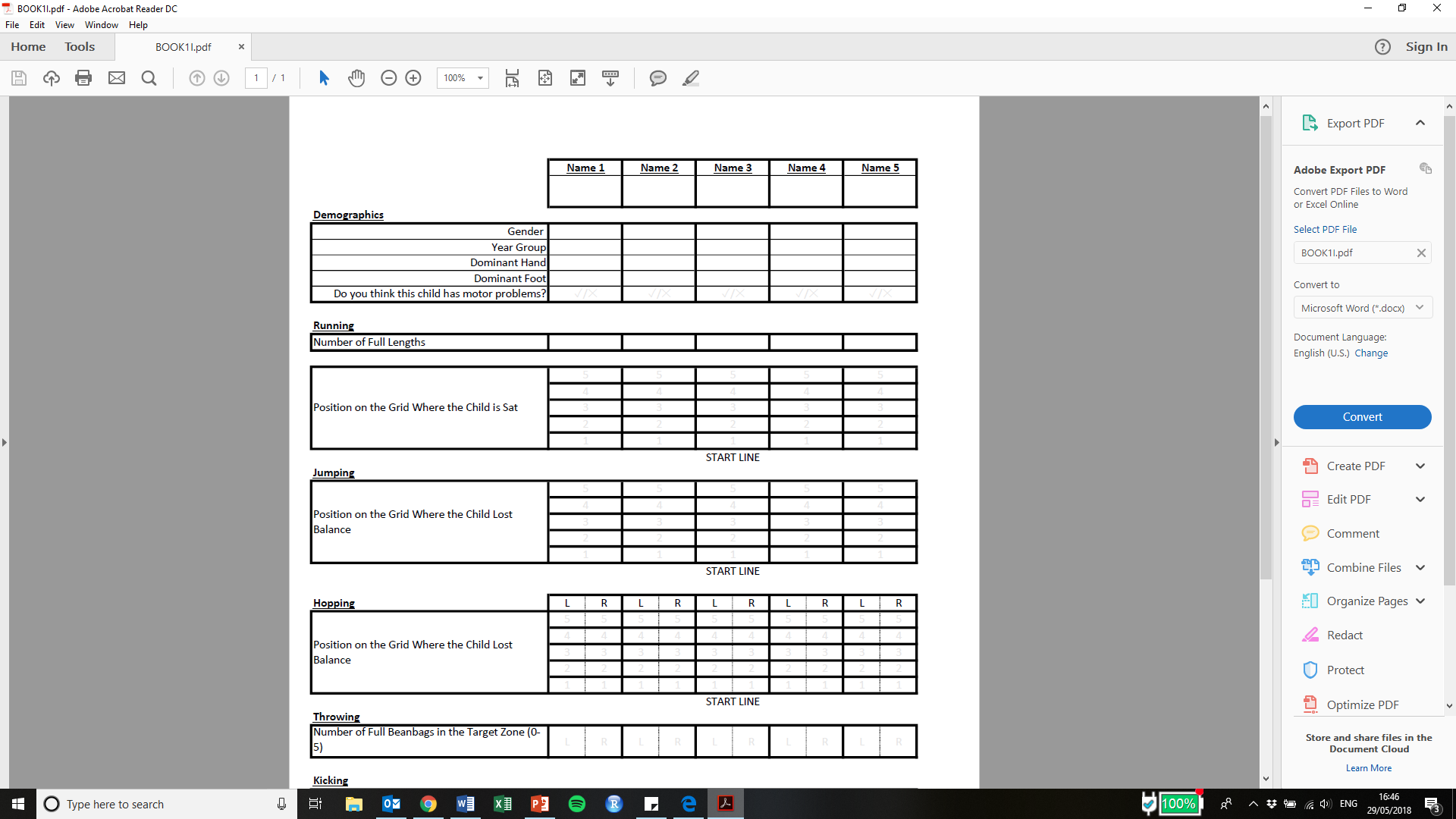
Scoring**

Children are scored by the number of **boxes** in their lane which are **filled** by their beanbags.

- Left handed score is noted in the L box, right handed score is noted in the R box for each child
- To get a point, the beanbag needs to be fully in their target box. If it is touching the boundary line but not crossing the outside edge, you can count this (see below for some examples)


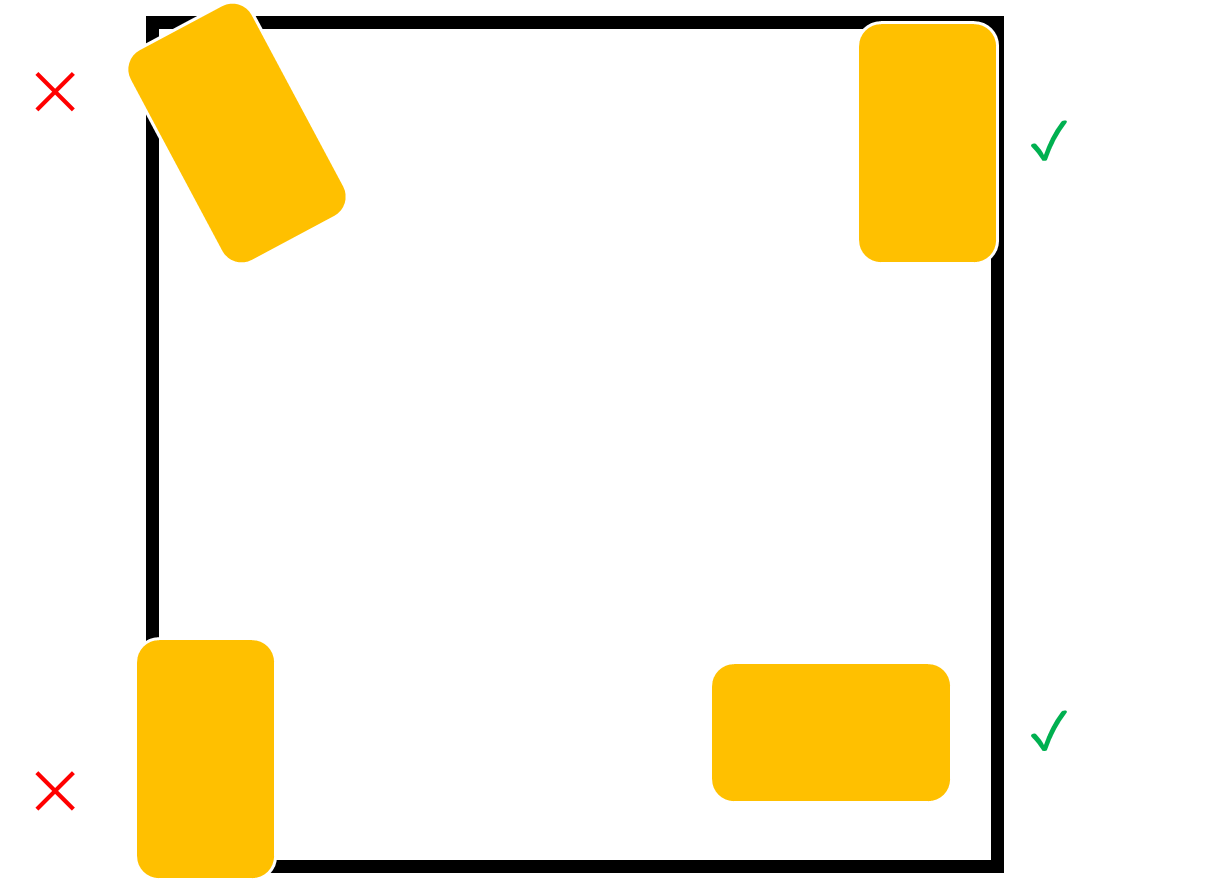


Kicking

**Set up**

- Each child needs 5 beanbags all that are the same colour
- Children stood next to one another on the grid should not have the same colour beanbags
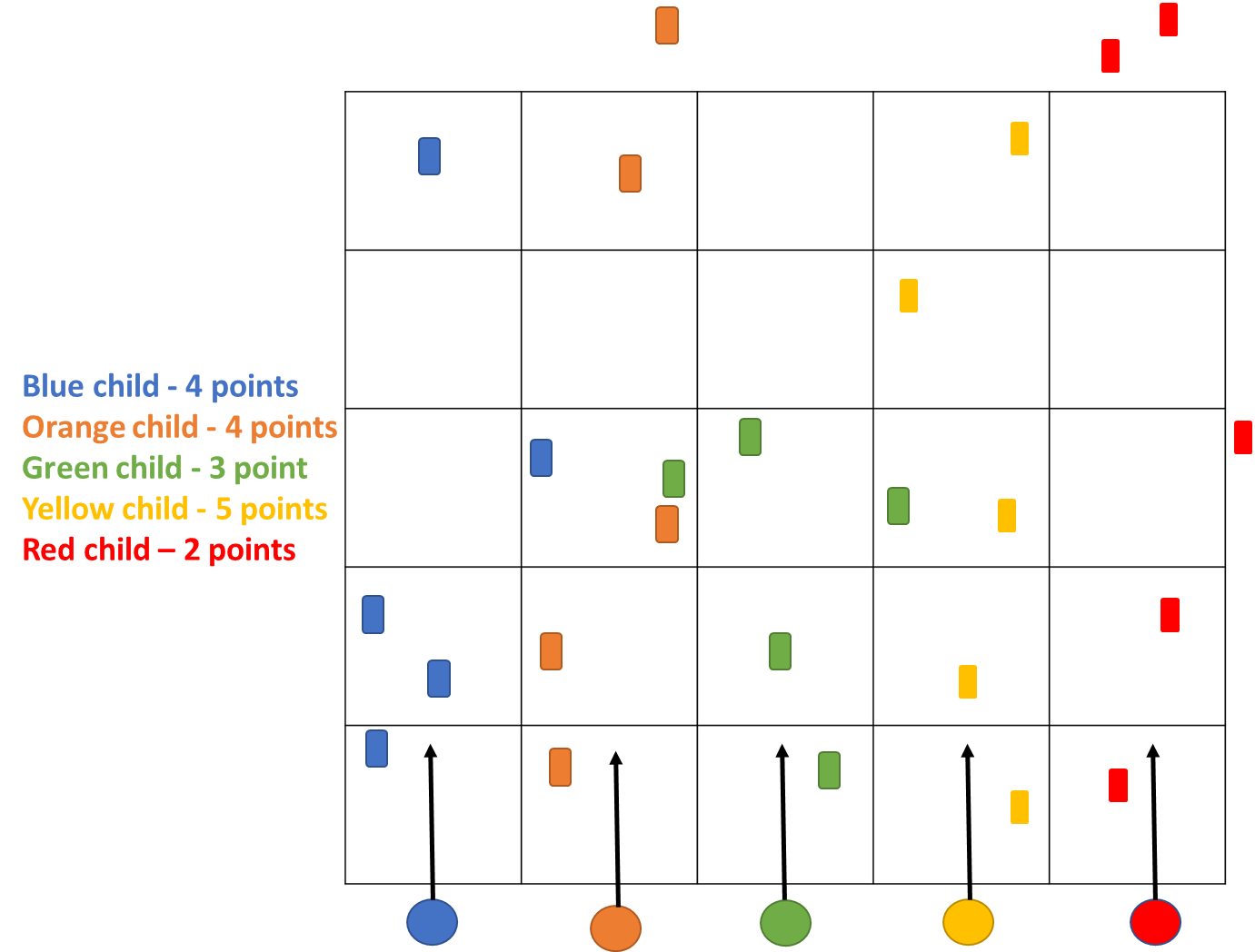


**Rules**

- Children aim to kick (along the floor) one beanbag into each box in their lane using whichever foot they want
- Children cannot change the foot they use during the activity
- Each child can only kick one beanbag at a time
- Points will only be awarded for beanbags that land in their lane
- Only one point can be awarded per box in their lane

**Demonstrate the task and explain the rules**

**Scoring**


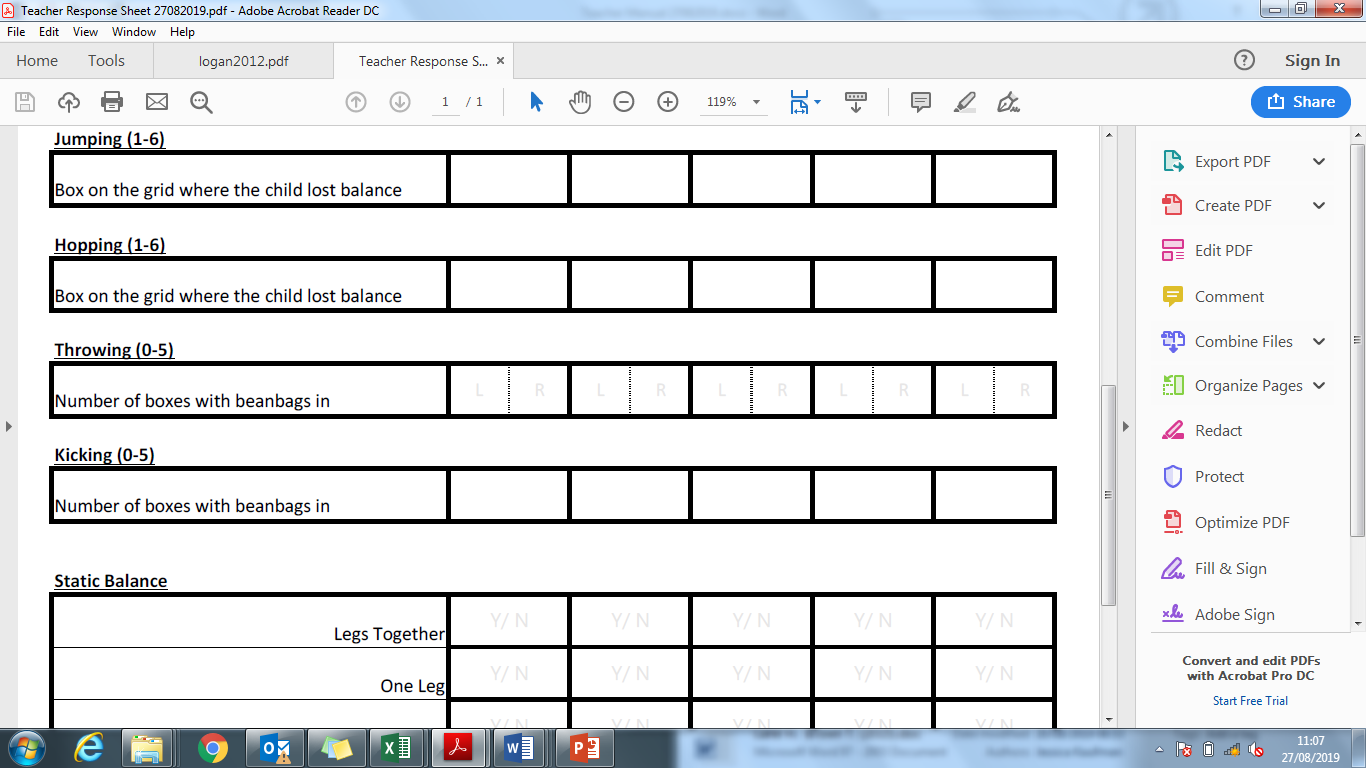
Children are scored by the number of **boxes** in their lane which are **filled** by their beanbags.

- To get a point, the beanbag needs to be fully in their target box. If it is touching the boundary line but not crossing the outside edge, you can count this (see below for some examples)


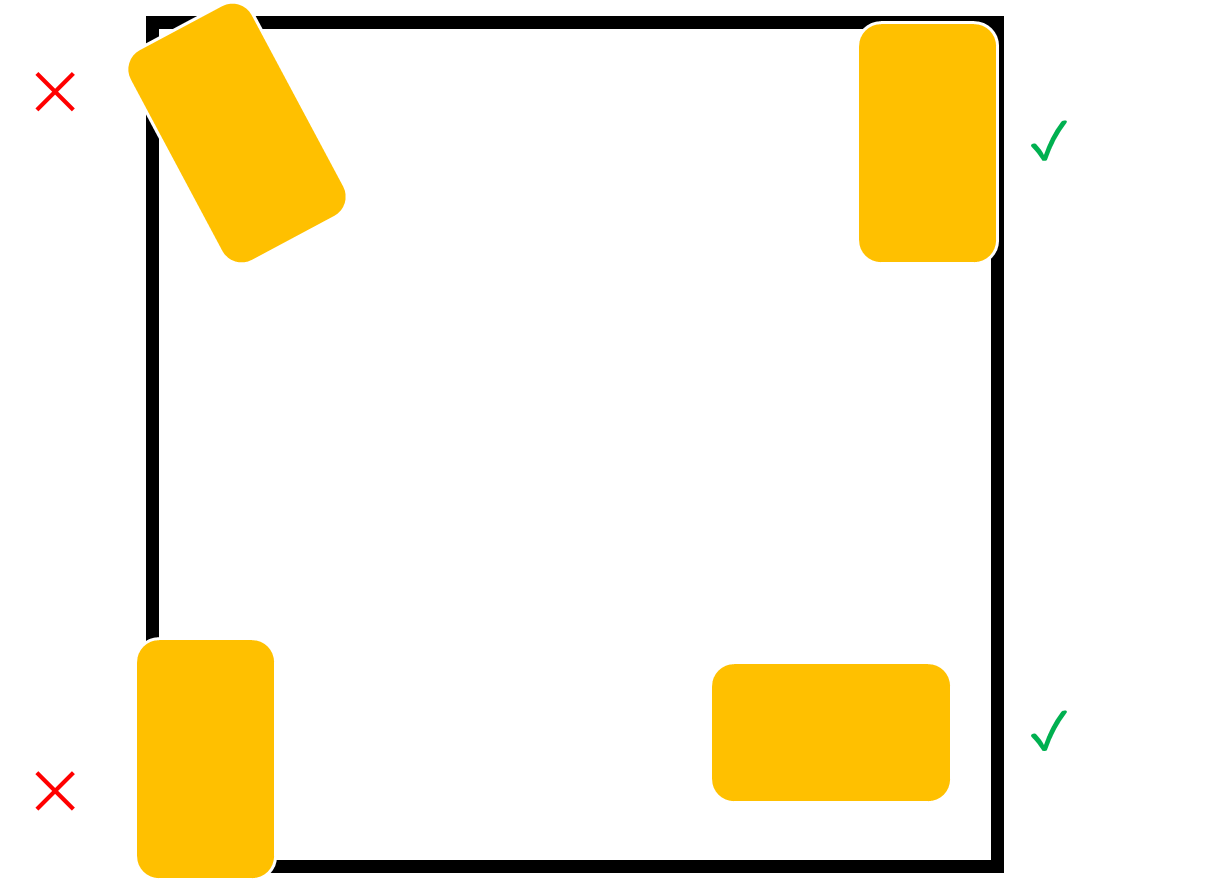


Static Balance

**Set up**

- Give each child one beanbag
- Line the children up next to each other, with enough space that children can swing their arms without hitting each other

**Rules**

- Balances 1, 2 and 4: children pass a beanbag around their body three times whilst holding a balance


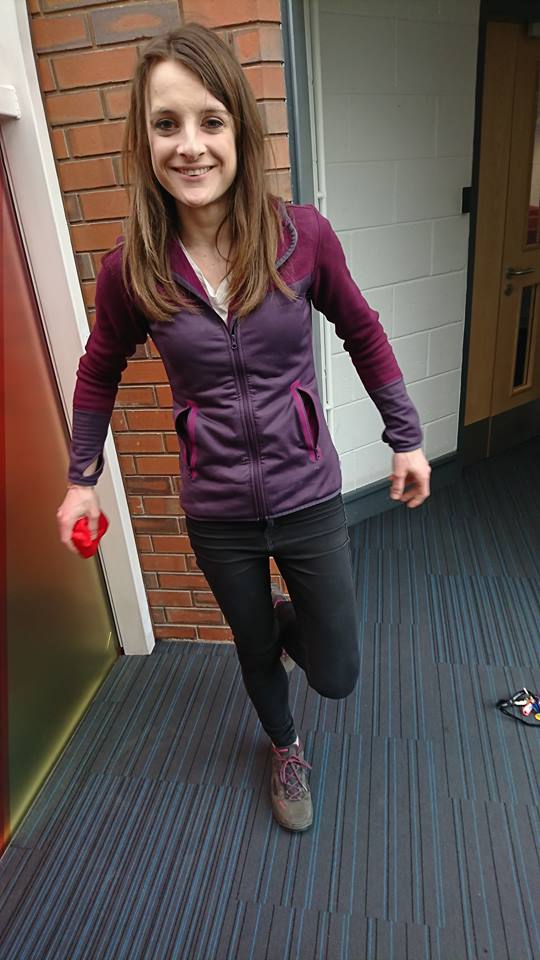

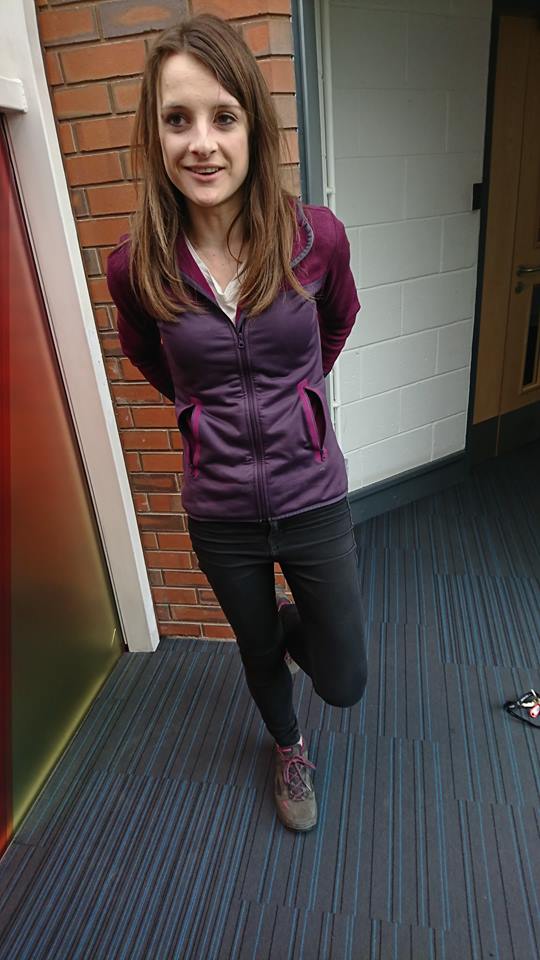

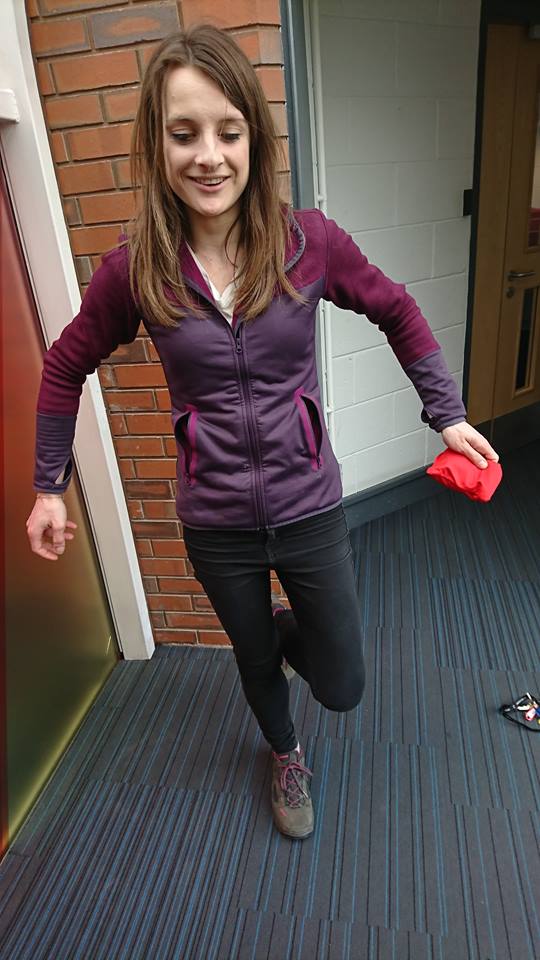

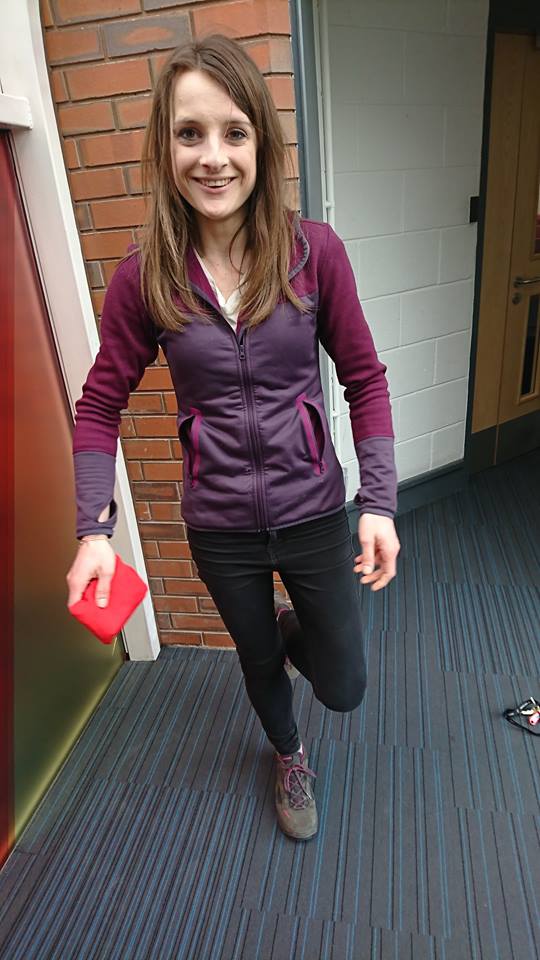


- - Balance 1 = feet together
  - Balance 2 = on one foot
  - Balance 4 = on one foot, with eyes closed
- Balance 3 - put each child’s beanbag on the floor in front of them, they should attempt to pick up the beanbag in front of them whilst balancing on one leg


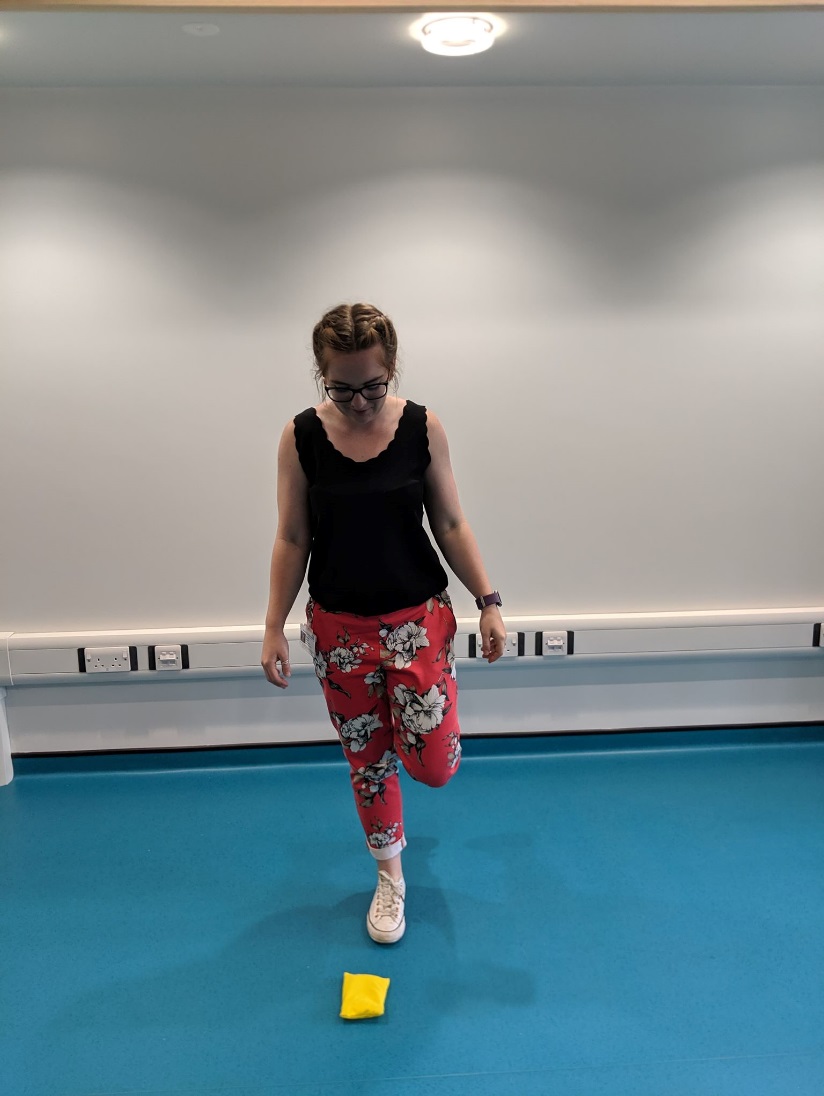

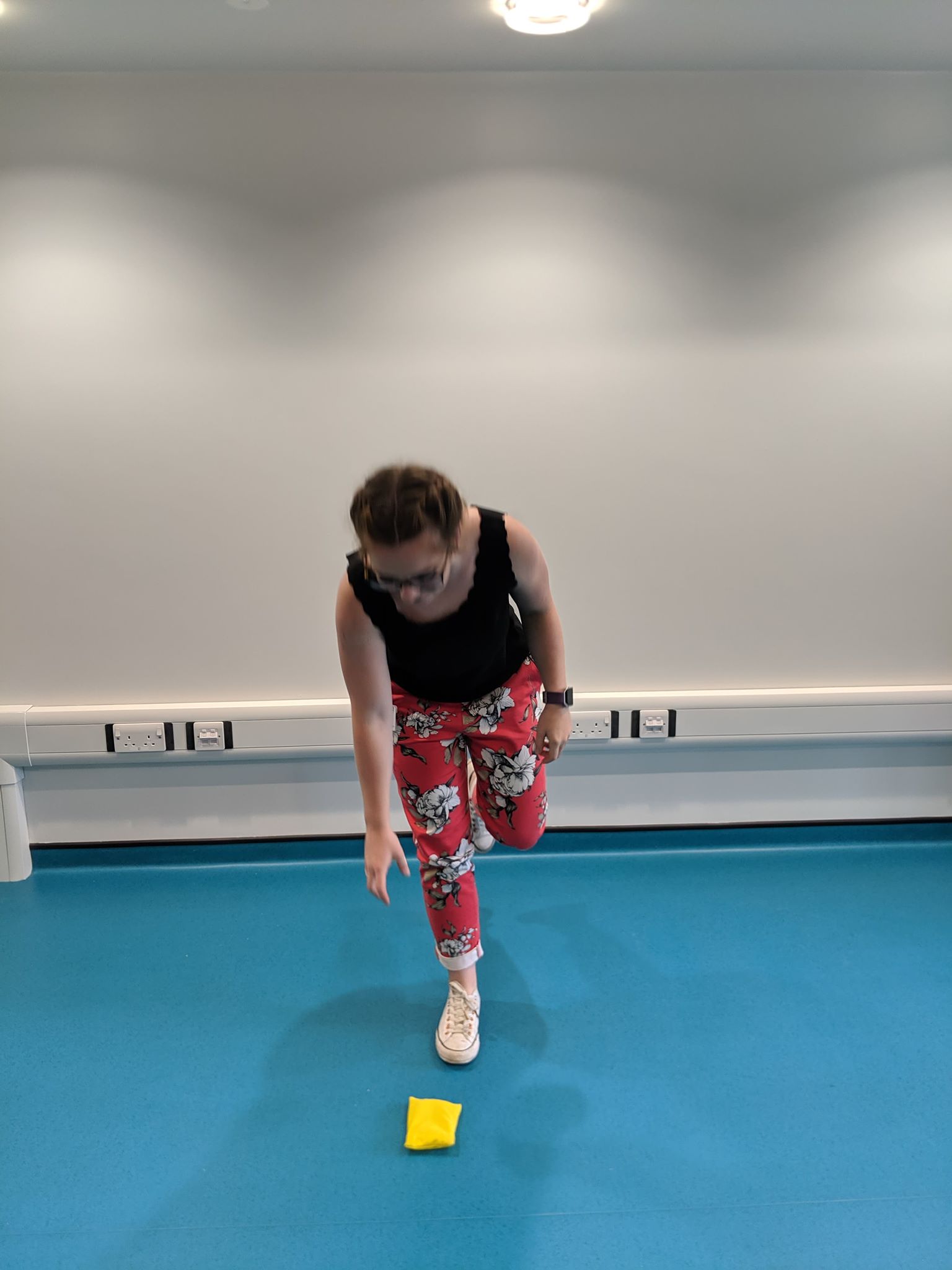

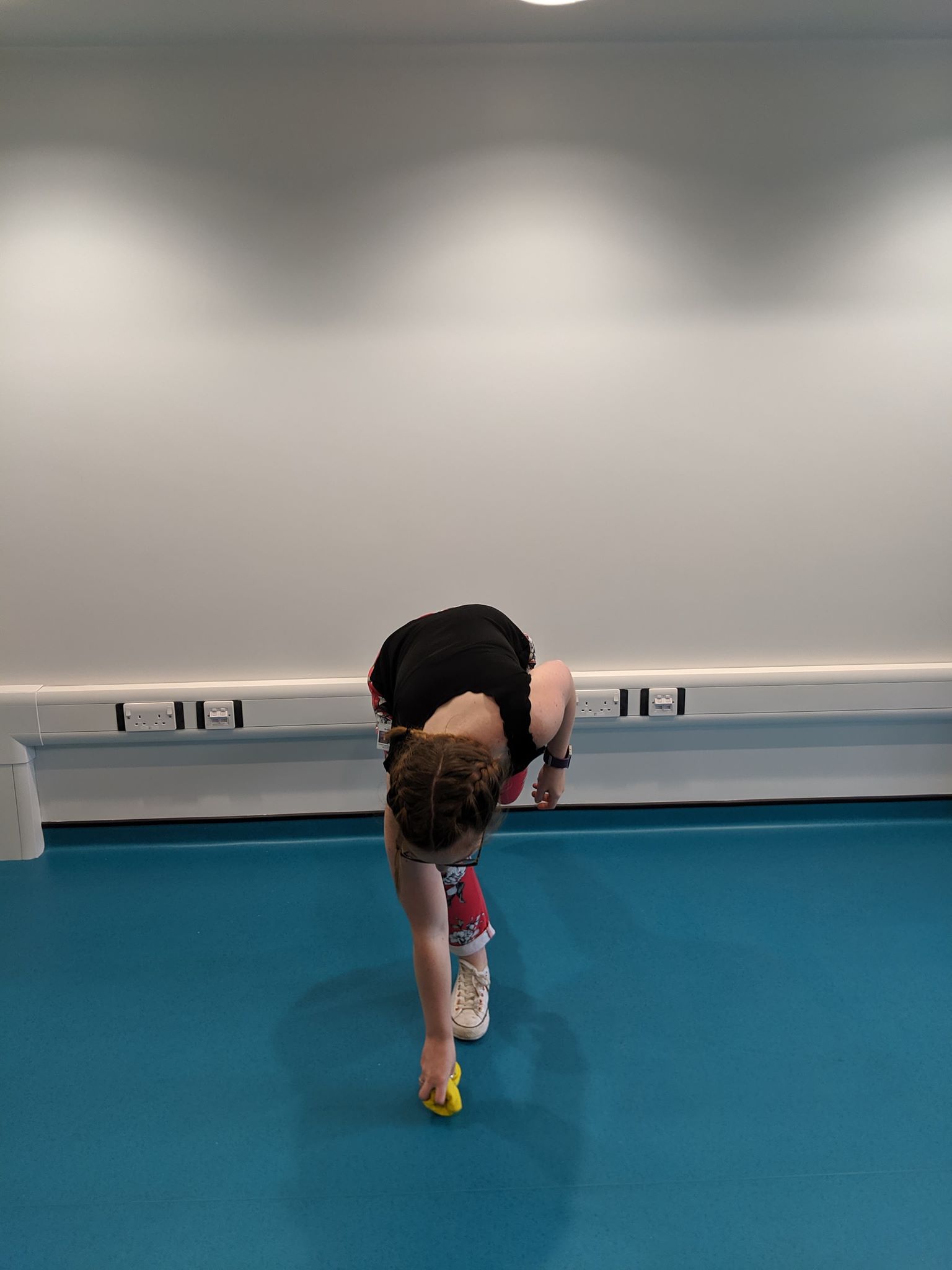

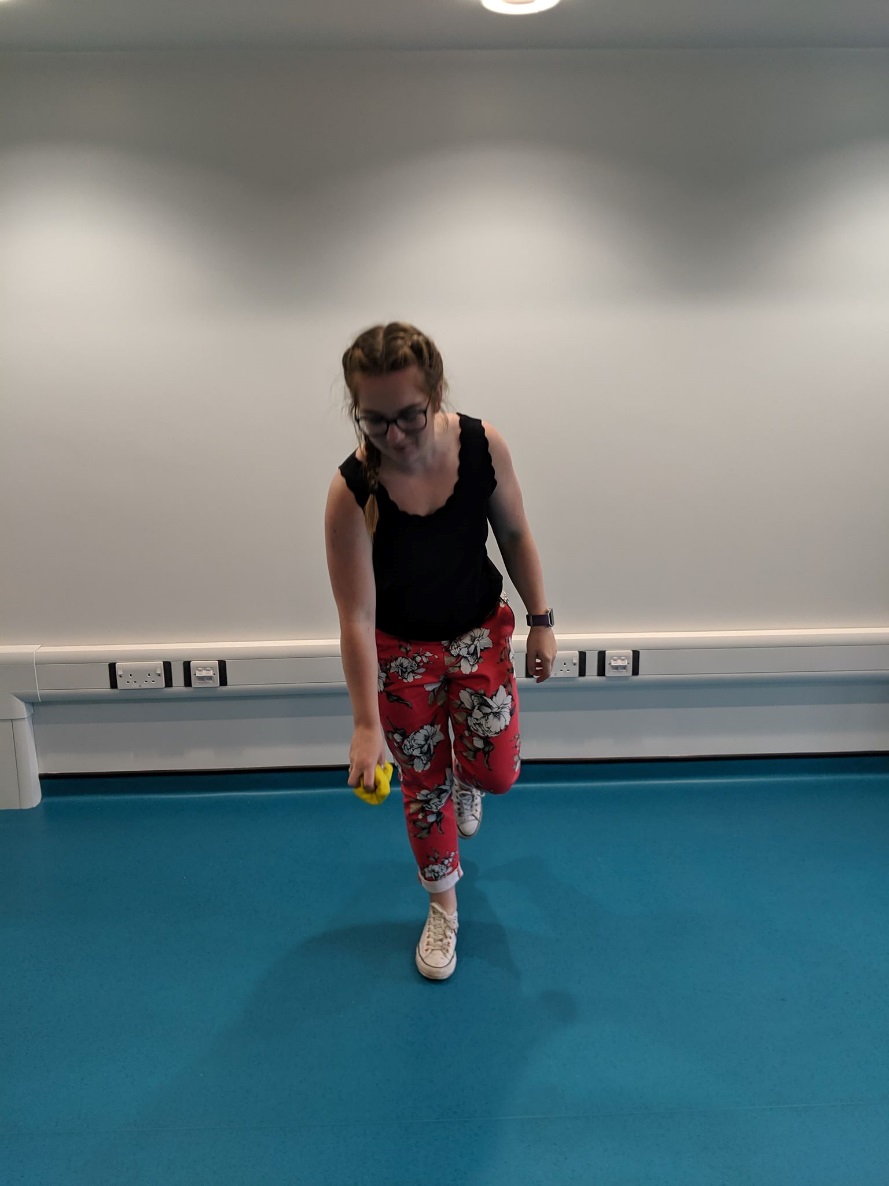

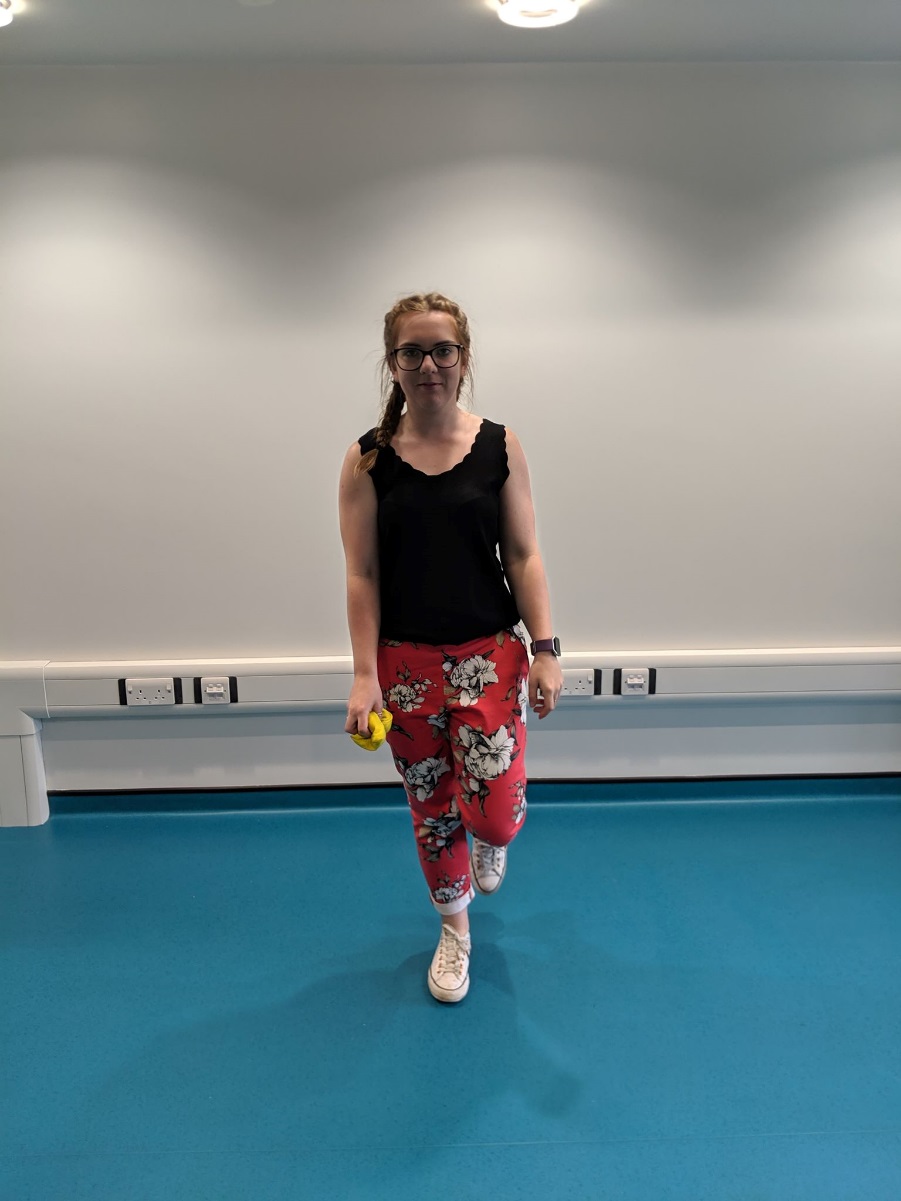


**Demonstrate each balance whilst ALL children are sat down and explain the rules, then get all groups to do balance 1 before moving onto balance 2. Count the number of rotations of the beanbags out loud.**

**Scoring**

Children are scored by whether they can successfully **complete** each **balance** (yes/no).


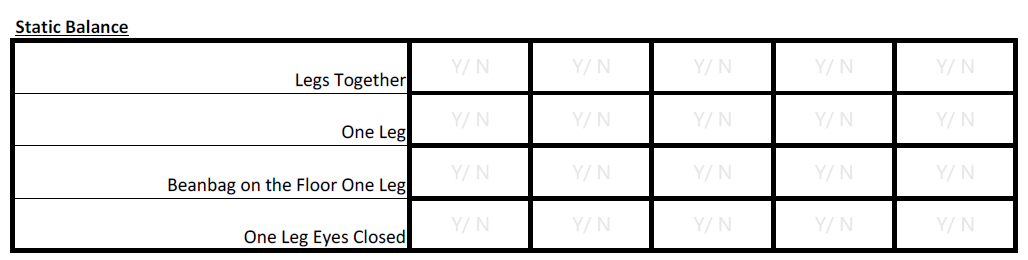


- Losing balance includes:
  - Dropping the beanbag
  - Not maintaining the balance position whilst passing the beanbag around their body
    - Wobbling is acceptable, but shuffling on their foot is not
  - Opening their eyes in an eyes closed balance
  - Putting their other hand down when picking up the beanbag in balance 4
- Place a Y (yes) or a N (no) on the response sheet to indicate whether each child successfully completed each balance
