# Supplementary material for: Developing and validating a school-based screening tool of Fundamental Movement Skills (FUNMOVES) using Rasch analysis
Source: PLoS One. 2021 Apr 16;16(4):e0250002. doi: 10.1371/journal.pone.0250002 (PMC8051776; doi:10.1371/journal.pone.0250002)
Supplement: S2 File — (DOCX) [file pone.0250002.s002.docx]

**S2 File. Teacher implementation fidelity checklist used in study 3.**

**Implementation Fidelity Checklist**

**Teacher ID: ______________________________________________________________**

**School: _____________________________________________________________________**

**Class Tested: _____________________________________________________________________**

**Preparation**

| Essential: | Preferable: |
| --- | --- |
| - Grid is set up | - Teams are located in the lane on the grid they refer to on the teachers sheet |
| - Teacher has a stop watch/ timer | - Physically show each team their lane |
| - Teacher has pens | Explain that the row they are sat in is their   team |
| - Teacher lines students up in their teams | Explain that each activity can earn their   team points |
| - Within each team, children are in the order they appear on the teacher’s sheet | Explain how to keep note of team scores on the score sheet |
|  | - Explain that they will receive no points if they do not follow the rules |

Comments:

________________________________________________________________________________________________________________________________________________________________________________________________________________________________________________________________________________________________________________________________________

**Running**

| Essential: | Preferable: |
| --- | --- |
| - Explain that they will be running for 15 seconds | - The first person to sit down when they say stop wins a bonus point |
| - Explain that they run from the start line to the back line as many times as possible | - Clarify with class for understanding of rules |
| - Explain that they should run as quickly as they can | - Ensure the children not running from each team are out of the way |
| - Explain that they must touch the line with their foot at both sides | - Teacher keeps a tally for each child whilst running |
| - Explain when they say stop, the children must sit down as quickly as possible | - Teacher uses a timer which beeps after 15 seconds |
| - Accurate demonstration of the task | - Explain that they will get no points if they don’t run all the way to the lines |
| - Teacher shouts stop after 15 seconds of running (time once to check) | - Explain that they must stay in their own lane when running |
| - Scores full lengths correctly (best judgement) |  |

Comments:

________________________________________________________________________________________________________________________________________________________________________________________________________________________________________________________________________________________________________________________________________

**Jumping**

| Essential: | Preferable: |
| --- | --- |
| - Explain that they must do small jumps from the first line to the second line and pause until they say go | - Ensure all children are sat down when explaining the activity |
| - Explain that they will do the same from the second line to the third line etc. | - Explain that they will get no points if they don’t follow the rules |
| - Explain that on each line they must land with both feet in the red/ coloured zone | - Explain that it isn’t a race, they are scored on how well they can jump - Explain that they should only start jumping when they say go |
| - Accurate demonstration | - Actively times 3 seconds |
| - Pauses children for approximately 3 seconds on each line | - Keeps all children sat down until their turn so they don’t get to practice beforehand - Explain that they cannot just jump from line to line |
| - Scores children correctly (best judgement) | - Explain that they must pause on the back line too |

Comments:

________________________________________________________________________________________________________________________________________________________________________________________________________________________________________________________________________________________________________________________________________

**Hopping**

| Essential: | Preferable: |
| --- | --- |
| - Explain that they must do small hops from the first line to the second line and pause until they say go | - Ensure all children are sat down when explaining the activity |
| - Explain that they will do the same from the second line to the third line etc. | - Explain that they will get no points if they don’t follow the rules |
| - Explain that they can hop on any leg but must not change legs during activity | - Explain that it isn’t a race, they are scored on how well they can hop |
| - Explain that they cannot just hop from line to line | - Actively times 3 seconds |
| - Explain that on each line they must land on one foot in the red/ coloured zone | - Keeps all children sat down until their turn so they don’t get to practice beforehand |
| - Accurate demonstration | Explain that they must pause on the back line too |
| - Pauses children for 3 seconds on each line | - Explain that when they say they should put one leg in the air, and only start hopping when they say go |
| - Scores children correctly (best judgement) |  |

Comments:

________________________________________________________________________________________________________________________________________________________________________________________________________________________________________________________________________________________________________________________________________

**Throwing**

| Essential: | Preferable: |
| --- | --- |
| - Explain that they should aim to throw one beanbag into each box in their lane | - Ensure all children are sat down when explaining the activity |
| - Explain that they should throw underarm | - Explain that they will get no points if they don’t follow the rules |
| - Explain that their foot should be behind the line when throwing | - Explain that they do not get more points for further away boxes |
| - Explain that they get one point for each box they fill in their lane | - Before each child starts, asks them to hold one beanbag in their right/left hand in the air to check they understand |
| - Explain that they will do the activity twice, once throwing all five beanbags with their right hand, once with their left |  |
| - Accurate demonstration |  |
| - Ensures children are throwing with the correct hand and re-sets if not |  |
| - Ensures children throw underarm and re-sets if not |  |
| - Physically checks beanbags which land near a line |  |
| - Scores children correctly (best judgement) |  |

Comments:

________________________________________________________________________________________________________________________________________________________________________________________________________________________________________________________________________________________________________________________________________

**Kicking**

| Essential: | Preferable: |
| --- | --- |
| - Explain that they should aim to kick one beanbag into each box in their lane | - Ensure all children are sat down when explaining the activity |
| - Explain that they should kick the beanbag along the floor, not out of hands | - Explain that they will get no points if they don’t follow the rules |
| - Explain that the beanbags should be behind the line before kicking | - Explain that they do not get more points for further away boxes |
| - Explain that they can use whichever leg they like to kick, but must not change leg | Explain that they get one point for each box they fill in their lane |
| - Accurate demonstration |  |
| - Ensures children are kicking along the floor and re-sets if not |  |
| - Physically checks beanbags which land near a line |  |
| - Scores children correctly (best judgement) |  |

Comments:

________________________________________________________________________________________________________________________________________________________________________________________________________________________________________________________________________________________________________________________________________

**Balance**

| Essential: | Preferable: |
| --- | --- |
| - Explains there will be a series of balance poses they need to hold | - Ensure all children are sat down when explaining the activity |
| - Balance 1: feet need to be kept together at all times | - Explain that they will get no points if they don’t follow the rules |
| - Balance 1: standing up straight you must pass the beanbag around your body with my count | - Removes beanbags from throwing and kicking tasks and hands each child a beanbag only when it is their turn |
| - Explain with my count: when I say 1, you pass it around your body the first time, 2 the second time etc. | - Explain that they can pass the beanbag around their body either way - Explain they should only pick up the beanbag when the teacher says so |
| - Ensures all children are sat down until it is their turn so they get no chance to practice | - Explain that they should only start passing the beanbag around their body when the teacher starts counting |
| - Balance 2: explains that they should do the same again but on one leg | - Explain that after the 3^rd^ rotation they should drop the beanbag in front of them, but maintain the balance until their beanbag hits the floor |
| - Balance 3: explains that they should place the beanbag in front of them | - Balance 4: explains they will get no points if they open their eyes |
| - Balance 3: explains that they should try to retrieve the beanbag, standing on one leg using one hand |  |
| - Balance 4: explains that they should stand on one leg and close their eyes to pass the beanbag around their body |  |
| - Accurate Demonstration for each balance |  |
| - Scores children correctly (best judgement) |  |

Comments:

____________________________________________________________________________________________________________________________________________________________________
