# Supplementary material for: Developing and validating a school-based screening tool of Fundamental Movement Skills (FUNMOVES) using Rasch analysis
Source: PLoS One. 2021 Apr 16;16(4):e0250002. doi: 10.1371/journal.pone.0250002 (PMC8051776; doi:10.1371/journal.pone.0250002)
Supplement: S1 Table — (DOCX) [file pone.0250002.s003.docx]

**S1 Table. Implementation Fidelity issues for study 2.**

| **Class Assessed** | **% Essential Criteria Met** | **Activity where essential criteria was not met** | **Criteria not met** |
| --- | --- | --- | --- |
| 1A | 100 | n/a | n/a |
| 1B | 94 | Jumping | Scoring was not deemed accurate by researchers |
|  |  | Balance | Did not say that feet need to be together for balance one  Did not count out the rotations of beanbags around the body so children were completing the balances at different speeds and were thus balancing for unequal amounts of time |
| 2A | 94 | Running | Didn’t tell children to run as quickly as they can  Didn’t say that they should touch the line at both sides with their feet  Didn’t demonstrate the task properly |
| 3A | 100 | n/a | n/a |
| 3B | 100 | n/a | n/a |
| 4A | 100 | n/a | n/a |
| 4B | 96% | Running | Scoring was not deemed accurate by researchers |
|  |  | Balance | Didn’t demonstrate balance four |
| 5A | 100 | n/a | n/a |
| 5B | 100 | n/a | n/a |
| 6A | 100 | n/a | n/a |
| 6B | 100 | n/a | n/a |
