# Supplementary material for: Developing and validating a school-based screening tool of Fundamental Movement Skills (FUNMOVES) using Rasch analysis
Source: PLoS One. 2021 Apr 16;16(4):e0250002. doi: 10.1371/journal.pone.0250002 (PMC8051776; doi:10.1371/journal.pone.0250002)
Supplement: S2 Table — (DOCX) [file pone.0250002.s004.docx]

**S2 Table. Implementation Fidelity issues for study 3.**

| **Year Group Assessed** | **% Essential Criteria Met** | **Activity where essential criteria was not met** | **Criteria not met** |
| --- | --- | --- | --- |
| 1 | 85 | Running | Teacher did not demonstrate (asked researcher to) |
|  |  | Jumping | Not explaining that they need to pause on the final line too |
|  |  | Hopping | Not explaining that they need to pause on the final line too  Teacher did not demonstrate (asked researcher to) |
|  |  | Balance | Teacher did not demonstrate (asked researcher to) |
| 2 | 100 | n/a | n/a |
| 3 | 92 | Set up | Teacher did not line up students in teams  Children were not lined up in the order on their response sheets |
|  |  | Hopping | Did not tell students that they couldn’t change legs during activity |
| 4 | 100 | n/a | n/a |
| 5 | 100 | n/a | n/a |
| 6 | 100 | n/a | n/a |
