# Supplementary material for: Developing and validating a school-based screening tool of Fundamental Movement Skills (FUNMOVES) using Rasch analysis
Source: PLoS One. 2021 Apr 16;16(4):e0250002. doi: 10.1371/journal.pone.0250002 (PMC8051776; doi:10.1371/journal.pone.0250002)
Supplement: Attachment — Submitted filename: Response to Reviewers.docx [file pone.0250002.s005.docx]

08/02/2021

Dear Dr Capio

**PONE-D-20-33626: ‘Developing and validating a School-Based Assessment of Fundamental Movement Skills (FUNMOVES) using Rasch Analysis’**

I would like to begin by thanking yourself and the reviewers for the extremely useful comments on the first submission of our manuscript. We are grateful for the opportunity to submit a revised version.

We have addressed all of the points raised by the reviewers, and feel the changes enhance the clarity of the manuscript. We have made all edits using tracked changes.

We have detailed how we have addressed each comment in turn overleaf – the page references refer to the anonymized document in ‘all markup’ format, unless otherwise stated.

Thank you again for your help with this submission, and we hope that this revised version is suitable for publication in PLoS One.

Yours sincerely,

Lucy Eddy

(On behalf of all co-authors)

Journal Requirements

1. Please ensure that your manuscript meets PLOS ONE's style requirements, including those for file naming.

Authors’ response: Thank you for highlighting inconsistencies with PLOS ONE’s style requirements. We have now amended the manuscript to align with these guidelines.

2. Please provide additional details regarding participant consent. In the ethics statement in the Methods and online submission information, please ensure that you have specified (1) whether consent was informed and (2) what type you obtained (for instance, written or verbal, and if verbal, how it was documented and witnessed). If your study included minors, state whether you obtained consent from parents or guardians. If the need for consent was waived by the ethics committee, please include this information.

Authors’ response: For this study we used written opt-out parental consent, where parents or guardians were asked to return a slip if they did not want their child to participate in the research. Children were also verbally assented on the day of testing (see page 8, lines 195-196). This was approved by the University’s Ethics Committee (reference: PSC-591). We will ensure that this information is included in the online submission.

3. We note that you have indicated that data from this study are available upon request. PLOS only allows data to be available upon request if there are legal or ethical restrictions on sharing data publicly. Please respond a) If there are ethical or legal restrictions on sharing a de-identified data set, please explain them in detail (e.g., data contain potentially identifying or sensitive patient information) and who has imposed them (e.g., an ethics committee). Please also provide contact information for a data access committee, ethics committee, or other institutional body to which data requests may be sent b) If there are no restrictions, please upload the minimal anonymized data set necessary to replicate your study findings as either Supporting Information files or to a stable, public repository and provide us with the relevant URLs, DOIs, or accession numbers.

Authors’ response: We have now provided a link to an anonymised dataset within the data availability statement.

4. We note that Supporting Information 1 includes an image of a patient. As per the PLOS ONE policy on papers that include identifying, or potentially identifying, information, the individual(s) or parent(s)/guardian(s) must be informed of the terms of the PLOS open-access (CC-BY) license and provide specific permission for publication of these details under the terms of this license. Please download the consent form. The signed consent form should not be submitted with the manuscript, but should be securely filed in the individual's case notes. Please amend the methods section and ethics statement of the manuscript to explicitly state that the patient/participant has provided consent for publication: “The individual in this manuscript has given written informed consent (as outlined in PLOS consent form) to publish these case details”.

Authors’ response: The two individuals that are photographed in supplementary material 1 have signed the appropriate consent forms. They are both of age to consent, and no identifiable information has been provided alongside the pictures. All scores on these pages are example scores, not the scores that these individuals scored.

Reviewer 1

5. Thank you for allowing me to review this interesting manuscript. I can see the authors have done lots of work to get to this stage. An easy to use universal screening motor test is important for teachers to find out children who needs help.

Authors’ response: We thank the reviewer for their appreciation of the value of universal screening in schools, and the work that has gone into the development of FUNMOVES. We have tried to address all of their concerns below (see responses to comments 6-10).

6. The children who might have motor delay were identified by the teachers, who were also the assessors using the FUNMOVES. This raises a question of bias as the assessors were not blinded. Furthermore, were these children "true-positives" with motor delay or other delay such as in language domain affecting their understanding of the instructions during the test? To test the discriminative ability of the FUNMOVES, it is essential to ensure that the children do have motor delay. I like the authors to comment this and point this out in their Discussion. Another psychometric study on children with motor delay would definitely prove the discriminative power of the FUNMOVES

Authors’ response: Thank you for raising this point. We agree that there are a number of issues with asking teachers to identify children with motor problems, and then consequently assess these children. We used this methodology as a *starting point* to give us an idea – broadly - as to whether FUNMOVES might be identifying the right children. During testing, however, it was very evident that teachers identified children with other difficulties, not just motor delay. One example was a child who had ASD that the teacher anticipated would perform badly on the tool, and was then surprised that they scored in line with their peers. This highlighted to us the power of using observational assessment tools over, for example, proxy report, in a school-based screening programme. We do agree that significant differences between teacher identified children and ‘typically developing’ children is not proof of discriminative validity, and future work needs to be done to establish such validity. This was not the purpose of the first phase of work reported in this manuscript. We plan studies of this type (though covid has affected our timescales) but we hope that the dissemination of the current work can allow others to conduct such studies. We have clarified the issues with teacher identification and the need for future work on discriminative validity within the discussion (see page 31, lines 559-567).

7. It is a pity that the FUNMOVES cannot be validated on children aged 4 to 5 years. It is essential to find those children who need help in earlier age than 6 years old due to their neuroplasticity. I understand to test a large of children in a short period of time is important in school settings. I wonder if testing these young children in smaller patches would help. This may be another follow-up psychometric study of the FUNMOVES for the authors.

Authors’ response: Thank you for this insightful comment. We agree that identifying children as early as possible is beneficial, and it is a shame that FUNMOVES on a group level is not suitable for this age group. In study one, where reception children were tested, this was indeed done in small groups (of five) children, that were taken out of class to complete the assessment. This was how the reception teachers at the school believed it would be most effective, and this indeed did work. Children were able to do the activities, and the additional staff required for this format was not problematic due to there being more support members of staff for this year group. We agree that a follow up study trialling the finalised version of FUNMOVES in this modified format for children aged 4-5 years old would be beneficial. We have included this suggestion for future research in the manuscript (see page 31, lines 568-576).

8. It is uncommon to present the person-item maps in the format of this manuscript. This makes the interpretation quite difficult for the problematic test items. Please consider to use the traditional person-item maps used in Rasch analysis studies.

Authors’ response: We thank the reviewer for this comment, but we are unsure what they are referring to within the manuscript. No person-item map was included in the first version of the manuscript. Below is the person-item map for study 3, for reference, which does not show issues with individual items (rather where the participants and items fell on the logit scale). We have now included this in the manuscript.

Figures 1 and 2 in the manuscript show category probability curves which demonstrate disordered thresholds for activities. Disordered thresholds occur when the response categories are in the wrong order. For example, children were never more likely to score 3 than 4 in the second study because balance 4 (one leg picking up a beanbag) is easier than balance 3 (one leg, eyes closed). These figures are the original RUMM figures, and the authors felt they were important to include as they show why changes to the scoring categories were necessary, appropriate, and resolved the issues. Figure 3 was included to give an overview of when we made changes and why (i.e. due to Rasch and/or implementation fidelity issues). This figure has now been removed and replaced with a table to avoid confusion.

9. I may have missed this- please state clearly if independent t-tests were performed in each study to test any difference between boys and girls. As mentioned by the authors in the Discussion, it is very unusual that both boys and girls performed equally well in these gross motor skills, especially in young age. Another reason for the non-significant results may be that those aged 4 to 5 years were not included in this study.

Authors’ response: Thank you for highlighting the absence of these details. An ANOVA was conducted for each study. We have added and clarified this information in the methods section (see page 12, lines 298-301). We agree that the non-significant results may indeed have occurred due to the lack of younger children within the sample as these differences are often more prominent in this group. We have included this point within the discussion (see page 30 lines 521-523)

10. I understand time is a main factor for a universal screening test. However the difference between dominant and non-dominant legs is very prominent in young children, especially for those with motor delay. However, the FUNMOVES does not explicitly indicate if both legs were tested or the children chose their preferred leg so as to save time. This may not be ideal when understanding child development and children with movement disorders. May the authors comment on this?

Authors’ response: We apologise for the lack of clarity regarding this issue within the manuscript. In study one, children were assessed using both legs (hopping & kicking) and arms (throwing), as we also believed that assessing both dominant (D) and non-dominant (ND) legs would be important. The Rasch analysis from this study, however, found that there was local dependency between hopping – D & ND, and kicking – D & ND. Local dependency demonstrates that the scores for D & ND legs for these activities were contributing the same information on ability and that by keeping both D & ND we were introducing redundancy to FUNMOVES, which was consequently affecting validity. Based on this analysis, the child was able to choose which leg they wanted to complete the hopping and kicking activities for studies two and three. Throwing continued to be included as both D & ND as we found no local dependency for this activity. We have clarified that children could choose which leg to hop on and kick with in the manuscript (see page 17, lines 359-360).

Reviewer 2

11. The manuscript under review describes a study to provide validation information of the FUNMOVES assessment tool. The author employed Rasch analyses in the paper, which seems an appropriate - yet perhaps slightly unusual - method to me. I have offered some comments from reading the paper below. I wouldn't consider myself as an expert in Rasch analyses, I apologize for any comments that I might have raised as a consequence of my lack of knowledge in this area.

Authors’ response: Thank you. We appreciate the time you have taken to review our manuscript. We have responded to each of the comments (12-21), in the order they appeared in the review.

12. I'm not knowledgeable in the UK situation, so I'm quite surprised that poor FMS is almost seen as a clinical health issue. I'm a little curious as to (i) whether this is applicable in other parts of the world, and (ii) what kinds of treatments patients would receive when they are referred. That said, these information are probably not extremely relevant to the main contents of this paper, so I'm happy if the authors decide to leave out such information in the paper.

Authors’ response: We thank the reviewer for this interesting comment. We are a little surprised that FMS is not seen as a clinical health issue elsewhere, given the links between FMS, physical activity and health that have been established within the literature (outlined in the introduction page 3 lines 54-56). Particularly in the UK, waiting lists for occupational therapy and physiotherapy services are over two years long, so there is most certainly a public health issue here. Whilst we cannot specifically comment on whether this is the case in other countries, it is noteworthy that the European Academy for Childhood Disability (EACD) provide guidelines on the diagnosis and treatment of Developmental Coordination Disorder^[1]^, a motor disorder that encompasses difficulties with both FMS and fine motor skills. The EACD guidelines are those followed by public health professionals in the UK and Europe when dealing with such difficulties.

13. There is a large difference between the approach of outcome-based tests (e.g., FUNMOVES) and "progress-based" tests (e.g., TGMD). I think this is a clear difference which some readers may not be aware of. I think the paper would benefit from some discussion and comparison between these methods - effectively, what are the pros and cons of each approach, specifically for the purpose of screening?

Authors’ response: Thank you for this constructive comment. We agree that the justification of product-based assessment for screening purposes is important. This was briefly included in the introduction in the first draft, but we have now expanded this justification (pages 5&6, lines 110-133).

14. Can the authors please provide a bit more context in terms of how PE classes are typically structured in UK? By knowing the frequency, duration, class size, number of teachers per class, etc of typically classes would help readers like me understand how feasible the tool can be applied. For example, from where I am from, PE classes are typically led by a single teacher, does that mean this tool may not be applicable? (to be fair, other tools wouldn't work well either) Also, I am rather curious as to what other students would be doing while the assessment is in progress. Would doing the assessment first/last give them a(n) (dis)advantage (e.g., if they could see what the test are beforehand)? Also, in the longer term, are the test results still valid when students do the test again a year later (assuming the screening is done annually), since they would have time to practice?

Authors’ response: This is a very insightful question. In the UK, typically classes have P.E. once a week for an hour in classes of approximately 30 children. The classes are often led by a single teacher, but sometimes teaching assistants are present. We decided to design FUNMOVES to be used by two members of teaching staff for: (i) speed; (ii) to help with behavioural issues. Moreover, a survey of primary school teachers (*n*=853) found that most (~78%) thought that they would be able to access a second member of staff, such as a teaching assistant, to enable them to undertake an FMS assessment^[2]^. As this assessment is designed to be a one-off process, accessing an additional member of staff should not be too burdensome for schools, particularly as teaching assistants are sometimes already present for P.E. lessons. We have now included a rationale for FUNMOVES utilising two members of teaching staff within the manuscript (see page 7, lines 172-176).

With regard to what the other children are doing whilst they are waiting, teachers are given the option as to whether or not they think their class would benefit from making FUNMOVES a competition between teams (lanes). For schools that have utilised the competition aspect, the children not participating have been responsible for keeping ‘team’ score and cheering on their teammates. Some aspects of this (e.g. cheering) was still observable in schools where teams were not keeping track of scores. But ultimately, children are sat waiting for their turn. We have tried to mitigate the effects of this in a number of ways. Firstly, all activities are quick, and take less than a minute per child to complete, so children are not waiting for too long. Secondly, when the teachers are grouping their children into fives by ability, they tested the highest ability group first, so that children that may benefit from additional demonstration received this from their peers. We agree that this may give some children an advantage and thus it will be important for future research to test the impact of testing order. We have included this point within the discussion (see page 32, lines 588-590).

15. What are the typical rates of students who meet the criteria for needing treatment in schools (or more specifically low SES schools)? Again, I feel this would provide context as to how "typical" the schools included are.

Authors’ response: Thank you very much for raising this point. It is difficult to give a figure as to how many children in low SES schools are struggling with FMS development as there have only been small-scale studies done thus far. Some UK research which recruited low SES samples suggested that 18.5% of children had not mastered any of the four FMS measured and 32% had only mastered one^[3]^, despite this being an expectation in the curriculum. In more ‘general’ samples, low levels of proficiency are also noted, with one study reporting 60% have poor FMS proficiency^[4]^, and another stating that only 2% of the sample had mastered all FMS measured^[5]^. We have provided more context for this within the manuscript (see page 30, lines 532-537).

16. Line 181: could authors be more specific as to what "sufficient power" means?

Authors’ response: Thank you for highlighting the absence of these details. This refers to having sufficient power to ensure measurement stability. With Rasch analysis, every time a set of items is calibrated on a different sample there is an expectation of slightly different results. One hundred and fifty participants ensures that differences are smaller and less likely to impact on overall validity. We have clarified this point within the manuscript (see page 8, lines 202-203).

17. I fully acknowledge the importance of having tools readily available to schools. But I am still a little skeptical as to how similar kicking a bean bag versus a ball would be. The roughness of the floor also greatly impacts how far the bag would travel. Without any practice at all, is this assessment still fair?

Authors’ response: We thank the reviewer for this insightful question. Firstly, it is important to note that we did not design the kicking activity to mimic kicking a football, nor was it designed to measure footballing ability. It was included to add an aspect of lower limb object control and balance to the assessment tool. Whilst we agree that this may not reflect how kicking is utilised in real-world situations, and that the surface may play a role in the distance a beanbag would travel, we are confident that this activity still measures the ability of interest for a number of reasons. Firstly, the ideal situation for FUNMOVES is in a sports hall, but these activities can be done outside, and have been trialled successfully in playgrounds by children. Secondly, this task will be novel (or at least largely unpractised) to all children, thus children will all have a similar level of experience on the task. The task requires children to modify their kick to adjust the distance in an attempt to fill all of the boxes in their lane. Children with better kicking ability should be better able to adapt to the requirements of the task quicker (demonstrating they possess good levels FMS). Finally, the kicking task met all Rasch psychometric properties and we can therefore be confident that it measures the construct of interest (i.e. aspects of FMS).

18. I believe the final "screening score" is the score by summing all the tests. But since each activity has different scores, is there a need to adjust or scale them? (or is that done by using logit scores?)

Authors’ response: When all data fit the Rasch model, it shows that the scores can be summed (regardless of the varying scales), as they are all measuring the same underlying construct of FMS. This relates to additive conjoint measurement^[6]^^[7]^^[8]^, and is one of the main strengths of using Rasch analysis – where other tools may sum activities arbitrarily, Rasch has shown that the summation of these scores is appropriate and valid, and therefore there is no need to adjust scores by activity.

19. I personally felt a written description of the modifications done would be helpful - potentially these could be added as additional columns in Table 1? That way it would be easier to see where things started and how they ended. (Figure 3 is not very clear as it doesn't describe the changes made)

Authors’ response: Thank you for this comment. On reflection, we agree with this suggestion. Rather than including this information in table one, at the beginning of the Rasch results, we have removed Figure 3 (the diagram of changes) and inputted this information as a table at the end of the third Rasch analysis so readers are not confused by the three iterations at the start of the results section.

20. Given this is a screening tool, can the authors provide any insight as to what threshold should be applied or used for identification of students that need to be referred? For example, can the authors provide the mean scores of students who were reported as having motor problems?

Authors’ response: Thank you for raising this point. For the purpose of the first three studies, and providing the schools with some insight into their pupils’ abilities, we used a threshold below the 15^th^ percentile cut off for each activity, based on each child’s performance compared to the rest of their year group. We do not think it would be appropriate to provide means for FUNMOVES in its current format. It is our aim to test a much larger sample of children in order to gather a normative dataset.

21. I feel the authors could provide some discussion in the merits of using Rasch analyses. For instance, why is it more appropriate than factor analyses in this situation?

Authors’ response: Thank you for this suggestion. The benefits of Rasch analysis are already included within the manuscript in the analysis section, but we have added additional benefits to this section too (see page 11, lines 260-272).

Reviewer 3

22. PONE-D-20-33626 presents the results of three studies that led to the development of FUNMOVES, a school-based assessment of FMS competency. The stated purpose was to develop an FMS instrument that could be feasibly used by teachers in physical education for a full class of children. The paper is comprehensive and is generally well-written. The use of Rasch modeling to examine construct validity is a clear strength. I do, however, have many concerns with both the FUNMOVES instrument and the manuscript

Authors’ response: We are grateful to the reviewer for recognising the clarity of the paper and the comprehensive analysis that we undertook for these studies. We have tried to address each of the concerns below (see comments 23-41).

23. I have spent a great deal of time contemplating the utility of this instrument. While I agree that feasibility within the school setting is important, I am conflicted whether this instrument provides useful information to the teacher about children with movement difficulties. The insistence upon a process-oriented approach may be more feasible, but does not necessarily provide the teacher with information to plan appropriate instruction – as would a process-oriented assessment. With the current focus on physical literacy in physical education (UK, Canada, USA), an instrument that address motor competence rather than skill seems more appropriate. I fully understand that priorities related to feasibility and process-orientation are likely diametrically opposed.

Authors’ response: The authors discussed at great lengths the pros and cons of product vs process assessments for universal screening in a school setting and, as the reviewer suggests, feasibility and process-oriented assessments do not go hand in hand. We chose to prioritise feasibility for a number of reasons. Firstly, it has been reported that there are a lack of P.E. specialist teachers in the UK^[9]^, many teachers have not received any training on FMS, and thus there is a lack of knowledge^[10]^. It is therefore likely that teachers would require extensive training to get them to a point where they would be able to, in real-time, accurately assess children’s FMS using process-oriented measures. With teachers already feeling time pressured^[11]^ , such extensive training is unlikely to be well-received or feasible for schools. This does mean, however, that teachers will receive less information about specific problems. Nevertheless, FUNMOVES was not designed to replace existing, more comprehensive measures of FMS, rather to screen all children so that children struggling can be referred for a more comprehensive assessment and/or support from services that are trained to deal with such issues (i.e. physiotherapy and occupational therapy).

The purpose of this screening tool is to identify children that would be missed using current referral routes (due to healthcare inequalities). With this in mind, it is important to note that children’s ability on product and process-oriented assessments of FMS are, in general, not very well correlated^[12]^. Occupational therapy and physiotherapy services in the UK use product-oriented assessments to measure motor skill ability, so there would likely be a large number of discrepancies between the children identified in screening programmes and those diagnosed within healthcare services if process-oriented measures were used in schools. This is likely due to the difference in focus (i.e. function vs form), which brings us back to the purpose of FUNMOVES. The purpose is to identify children struggling with FMS development that are unable to participate in physical activity and thus lead a healthy and fulfilling life, and in order to participate, children do not need to follow expert movement patterns.

We have included a paragraph in the introduction discussing the pros and cons of product vs process oriented assessments and justifying product-oriented assessments for this purpose (page 5 & 6, lines 110-133).

24. The study is strong with regards to the use of Rasch modeling to provide evidence of construct validity for the instrument. However, I question the face and content validity of the instrument that proceeds construct validity. That is – does this instrument truly measure FMS competence? The most troubling item is running. With the product-oriented assessment of distance traveled over 15 seconds, the instrument is measuring speed, not motor competence. Speed is highly correlated with a variety of biological factors that do not pertain to FMS competence.

Authors’ response: We agree that a straight line run would be highly reliant on speed. We were careful, however, to ensure that this was not the aspect being assessed when designing the running activity. The working group included psychologists with expertise in motor skills as well as occupational therapists and physiotherapists with extensive experience in measuring children’s motor ability (for full details of working group see response to comment 26). There were numerous meetings amongst the group to discuss what should be included within FUNMOVES. As stated in the manuscript, the development of activities followed two other pieces of work, one of which was a systematic review of observational FMS assessment tools. The working group carefully considered these assessment tools alongside the feasibility criteria from the second piece of work, a teacher questionnaire, and concluded that current measures were not suitable for use in universal screening programmes in schools.

The working group then looked at what FMS were regularly measured in assessment tools, how they were measured, and discussed the best way to measure these skills in a group setting, in schools with limited equipment. There were a number of assessment tools highlighted in the systematic review that evaluate product-oriented outcomes for running including the BOT-2^[13]^, FMS Polygon^[14]^, and the Stay in Step Screening Test^[15]^, all of which included a timed shuttle run. We wanted to modify these activities to ensure that performance was not solely reliant on speed and/or other biological factors. Firstly, we chose a short timeframe (15 seconds) so that fitness did not have too much of an impact upon performance. Secondly, we ensured that we were not measuring straight-line speed by designing a running activity that incorporated agility, which is noted as a key aspect of the primary school P.E. curriculum in the UK^[16]^. As such, children are not running at full speed for the duration of the activity. For each length they run, children are required to accelerate, and slow down to enable them to turn on the line to accelerate again and re-start the process. Finally, the Rasch analysis showed that this item met all psychometric properties necessary for fit to the model. Not only this, but the final version of FUNMOVES was found to be unidimensional and, as such, suggests that running is measuring the same overarching construct as the other activities within the battery (i.e. FMS), rather than, for example, speed. We are confident that the thorough background work and the comprehensive testing and modifications using Rasch analysis ensure that FUNMOVES has strong face, content and construct validity.

25. Page 3 – Line 64: Are the authors referring to children with Developmental Coordination Disorder? If so, a clearer statement is needed. More introduction related to DCD and FMS development would also be warranted. If not, it would be useful for the authors to then operationalize “struggling with FMS development”.

Authors’ response: We apologise for the oversight in the introduction. This sentence does refer to diagnosis of DCD, this has now been clarified in the manuscript (page 3, lines 63-66).Whilst we agree that this needed to be clarified, we believe including more research on DCD in the manuscript will dilute the ‘story’ for a number of reasons. Firstly, although FUNMOVES will (hopefully) highlight children with DCD, there are a number of other children struggling with FMS that it will also hopefully highlight. This is due to the stringent criteria for DCD meaning that children with intellectual disabilities, visual impairments and neurological conditions will not receive a diagnosis. Additionally, it is likely that there are a number of children who have competent fine motor skills, but who are unable to perform FMS adequately. Therefore we do not want FUNMOVES to only identify children with DCD, rather all children that are struggling with FMS development.

26. Page 6 – Section 2.1: The authors’ self description of the working group does not include anyone with expertise in motor development. Many of my concerns are related to the appropriateness of FUNMOVES to measure FMS competence, which I believe would be shared by others in the field.

Authors’ response: We apologise for any confusion with regard to the description of the working group within the manuscript. The working group was carefully convened for their expertise. The group included two psychologists that specialise in motor development, who, between them have over 100 publications spanning how motor skills are executed, and assessed, as well as correlates of motor ability, and interventions to improve proficiency. They are regular keynote speakers at international motor skill conferences. Moreover, they have relevant experience in developing a motor skill assessment tool for children that is used widely for major international programmes of work (e.g. the evaluation of London’s Ultra Low Emission Zone). One of the group sits on a number of government groups (including within the UK’s Department for Education) in order to provide advice on motor skill development in children.

Similarly, the team includes a physiotherapist and an occupational therapist, who have extensive experience of physically assessing children’s motor ability and a wide range of research experience. This experience includes designing new assessment tools for motor function (upper limb functionality for children with cerebral palsy), and designing and implementing motor skill interventions in a school setting (handwriting and FMS). There is also a behavioural epidemiologist on the team whose expertise is physical activity, but also has experience of using behaviour change theories to (i) evaluate interventions (ii) understand barriers and facilitators to new initiatives. All authors also have extensive experience working in schools, and with educational bodies, such as the Department for Education, which brings knowledge and understanding of: (i) what schools want; (ii) what would be feasible for schools; and (iii) how initiatives can be best implemented in these settings. The expertise of the working group has now been clarified within the manuscript (see page 6, lines 149-152).

27. Page 8 – Sections 2.3.1, 2.3.2 & 2.3.3 – For each of these sections, the last sentence describes the number of children in each sample identified with motor difficulties/problems. Please include more information on how this was determined.

Authors’ response: Prior to testing, we asked teachers whether they thought each child had difficulties with their motor skills. Teachers were given guidance as to the signs of a child with motor difficulties at the training session. This has been clarified in the manuscript (see page 9 lines 207-210).

28. Page 8 – Section 2.3.3 – How does the unreliable data from the school relate to the overall feasibility of this instrument?

Authors’ response: Thank you for raising this important point. We did not specifically evaluate feasability within the three studies, as it was outwith the scope of this work. The commentary in the discussion around feasibility is linked to previous research^[17],^^[18]^, and the guidelines set out in previous research for feasibility of assessments in school settings. There are plans to evaluate feasibility in a more formal way (e.g. Focus groups) but covid has affected our ability to undertake such further studies to date. We have included a section in the discussion to highlight the problem with the unreliable data from one of the schools, and the need to formally evaluate feasibility (see page 32, lines 580-588). Whilst we agree that unreliable data may infer issues with feasibility, it is important to note that the teachers themselves were able to implement and score the activities with the main problem being that the school hadn’t timetabled sufficient time for the assessments to take place. Thus, half of the scores were researcher-led during break-time and lunchtime, which is not how FUNMOVES should be delivered.

29. Page 8 – Section 2.4 – How were teachers trained in studies 1 and 2? This section only refers to study 3.

Authors’ response: We apologise for the confusion caused. The teachers were trained in the same way for all three studies, as described later in the manuscript on page 10, lines 239-245. The manual that is attached as supplementary material was the manual used in study three. Manuals were used in studies one and two as well, but they changed due to changes with the activities throughout development. We thought it would be most appropriate and useful to attach the latest version of the manual.

30. Page 9 – Section 2.5 – What proportion of children in study 1 were in the reception year (4-5yo)? How is this reflected in the information provided in section 2.3.1 and then the results in section 3.1?

Authors’ response: Thank you for highlighting the absence of these details. There were 48 children in the pilot study which were from Reception year, which is now included in the manuscript for clarity (page 10, line 236). The reception children were included in the analysis of study one (both Rasch and implementation issues). No issues were unique to just reception, or just years 1-6.

31. Page 10 – Section 2.6 – In addition to the Rasch model and ANOVA, how was feasibility analyzed?

Authors’ response: Thank you for raising this point. For this study, feasibility was not specifically measured. FUNMOVES was evaluated in the discussion against criteria outlined in previous research for feasibility of school based assessments^[19]^^[20]^. We understand, however, that this is not sufficient to say that FUNMOVES is feasible for use in schools. We have edited the discussion to reflect that this is a ‘first step’ and that it will be important to undertake this further work to establish feasibility (see page 27, lines 486-489)

32. One overall source of confusion in the results was the modifications section in each study. Does this section reflect changes that were made following the study, that would then be reflected in the instrument used in the subsequent study? For example, does section 3.1.4 describe modifications that were made following study 1 and then used in study 2? If so, I am concerned about the changes made to scoring in section 3.3.3 that led to the final instrument, but were not tested in the field. Even if I am incorrect, more clarity in the-se sections is needed.

Authors’ response: The reviewer is correct that the modifications following the analysis reflect changes to FUNMOVES that were then tested in the subsequent study. One limitation of this three-part study is that we have not yet tested the final scoring format. Unfortunately COVID has limited our access to schools and thus we have not been able to test this ‘in the field’ yet. After two iterations of development and Rasch testing, we felt that we had not yet achieved optimal performance in scoring for all items. After the third Rasch analysis, we made adaptations to the scoring of two items (jumping and hopping) to ameliorate disordered thresholds. The fit to the Rasch model with these modifications in place makes us confident that the new scoring thresholds will be appropriate, particularly as the activity itself is not being fundamentally changed, only the scoring. Nevertheless, it will be important to test this in a subsequent study. We have included this as a limitation within the paper (see page 31, lines 548- 554). It is also important to note that three rounds of Rasch analysis is unusual, and is far more comprehensive than the development of many other outcome measures.

33. Table 1 – I was confused by the inclusion of the original FUNMOVES items it Table 1. I believe it would be more useful to present the final version of the instrument within the manuscript. The original version could be included as another supporting information document.

Authors’ response: We agree that the final version of FUNMOVES would be useful to present in the manuscript but we feel that knowledge about the activities is useful for interpretation of the results throughout. Therefore, we have retained Table 1, but also included a table (3) which details each of the three iterations of FUNMOVES, as well as how and why they were modified (i.e. Rasch & implementation issues highlighted at each stage). We have also removed figure 3 (overview figure) to avoid duplicating information.

34. Fidelity – In each of the three studies, it would be useful to report either a percentage of classes that met full compliance and/or the average compliance (%) across all classes. What threshold did the study team determine was “acceptable” fidelity for a class? For example, was 85% fidelity for class 1 in study 3 sufficient?

Authors’ response: We are grateful for this useful suggestion. We have included average compliance across classes for studies two (page 17, line 366) and three (page 18, line 400) within the text (the studies which we are able to report this for). With regard to an ‘acceptable’ level of fidelity, we agree that 85% is potentially not high enough. It is, however, important to note that teachers were corrected before any child was tested, so that this did not impact the results. It was important that these activities were being implemented as intended to ensure that the final battery (and the reasons for the modifications up until that point) were as psychometrically sound as possible. We do agree that fidelity is important, and that the 85% fidelity reflects improvements that need to be made to the teacher training and/or the manual intended to assist teachers with the implementation of FUNMOVES. It will, also be important to establish whether teachers can, in fact, deliver FUNMOVES as intended, without the support of a researcher, as this will be essential if it is to be used for universal screening purposes. This is something we are looking to do in future studies (and publication of this work will allow others to explore such issues), and has now been highlighted within the discussion (see page 32, lines 576-588).

35. Line 392 – The authors need to make a much stronger for argument for why a PSI below 0.7 is still acceptable for a screening measure. The rest of your construct validity evidence is solid, but this point appears to be arbitrary.

Authors’ response: Thank you for highlighting the lack of clarity in this section. Below is a person-item map (now included in the paper, see Fig 3) which shows where both the sample (pink) and activities (blue) were located on the logit scale for the final study. As can be seen, many of the participants in this sample were above average ability (with average being 0 on the logit scale), and showed little variance in ability. This is perhaps unsurprising, given the demographics of the school (higher SES) and the fact they already had many physical activity initiatives in place. This does, however, impact upon the PSI value. As there are only a few measurement points where the bulk of abilities were located, this explains why the PSI was lower than we would have liked. There are a large number of measurement points below the ability of the participants in study three that were not being utilised, but would be utilised in a school that had more children with a greater variation of motor ability. The scoring thresholds are spread out sufficiently along the scale range, which suggests that the scale will capture children across the full range of ability and, more importantly, identify the group of children that we wish to screen i.e. those with poor FMS. Subsequent research is planned to evaluate responsiveness and on a broader range of targeting, that will include children known to have poor FMS ability (as identified by a well-established measure of FMS). We have included the PSI <.7 as a limitation in the discussion (see page 31, lines 554-567).

36. In general, I encourage the authors to soften their discussion of the findings. The consistent point of the discussion is that robust psychometric evidence for FUNMOVES has been provided. However, only evidence for feasibility and construct validity have been provided – and the final PSI less than 0.7 is certainly not evidence of robust or strong validity. There are many other psychometric properties that were not examined that are highly relevant to the utility of this instrument, including test-retest reliability, rater reliability (due to the need to assess up to five students simultaneously), content validity, and criterion validity

Authors’ response: We apologise for any over-emphasis of the results. We have now softened the discussion of these findings, and highlighted that these three studies were only a first step in the process (see page 32, lines 569-572). We agree that other aspects of validity and reliability will need to be established, and there are studies planned to do so.

37. It is also relevant to discuss the feasibility of FUNMOVES within the context of researcher assistance. In all three studies, the researchers were part of the process that made measurement feasible. An example of this issue is the feasibility data in study 3 where the researcher provided the demonstrations instead of the teacher. True feasibility of teachers using FUNMOVES will still need to be established without outside assistance

Authors’ response: Whilst we agree that this will be important to evaluate in future research, it was essential when developing the activities and validating the assessment tool that the activities were implemented correctly. This was a crucial first step, but we have highlighted the need for feasibility to be established without researchers present in future studies (see page 32, lines 580-583).

38. The analysis shows that FUNMOVES is capable of detecting differences between children that were pre-determined to have movement difficulties by the teacher and those that do not. This is certainly a useful piece of validity evidence. However, no information or evidence was provided for how FUNMOVES can be used to identify children “struggling with FMS development”. Evidence of diagnostic accuracy is critical for the instrument to meet the intended purpose.

Authors’ response: We agree that significant differences between teacher identified children, and ‘typically developing’ children is not proof of discriminative validity, and that future work will need to be done to establish this. We have clarified the need for future work on discriminative validity within the discussion (see page 31, lines 562-567).

39. No limitations are provided in the discussion.

Authors’ response: We apologise for the oversight. Limitations were interspersed throughout the discussion but have now been compiled together in a section at the end of the discussion (see pages 31-32, lines 548-590).

40. Please review the full manuscript, including supporting information documents, for editing.

Authors’ response: We thank the reviewer for this suggestion, we have now checked through the manuscript and supporting information and made any necessary changes.

41. Please review the reference list for errors and consistency.

Authors’ response: We thank the reviewer for this observation, we have now checked the reference list and amended it for errors.

1. Blank, R., Barnett, A. L., Cairney, J., Green, D., Kirby, A., Polatajko, H., ... & Vinçon, S. (2019). International clinical practice recommendations on the definition, diagnosis, assessment, intervention, and psychosocial aspects of developmental coordination disorder. Developmental Medicine & Child Neurology, 61(3), 242-285.
2. L.H. Eddy, L.J.B. Hill, M. Mon-Williams et al. Fundamental Movement Skills and their Assessment in Primary Schools from the Perspective of Teachers, 04 June 2020, PREPRINT (Version 1) available at Research Square [https://doi.org/10.21203/rs.3.rs-32875/v1]
3. Duncan, M. J., Roscoe, C. M., Noon, M., Clark, C. C., O’Brien, W., & Eyre, E. L. (2020). Run, jump, throw and catch: How proficient are children attending English schools at the fundamental motor skills identified as key within the school curriculum?. *European Physical Education Review*, *26*(4), 814-826.
4. Tyler, R., Foweather, L., Mackintosh, K. A., & Stratton, G. (2018). A dynamic assessment of children’s physical competence: The dragon challenge. *Medicine and science in sports and exercise*, *50*(12), 2474.
5. Farmer, O., Belton, S., & O’Brien, W. (2017). The relationship between actual fundamental motor skill proficiency, perceived motor skill confidence and competence, and physical activity in 8–12-year-old Irish female youth. *Sports*, *5*(4), 74.
6. Luce, R. D., & Tukey, J. W. (1964). Simultaneous conjoint measurement: A new type of fundamental measurement. Journal of mathematical psychology, 1(1), 1-27.
7. Perline, R., Wright, B. D., & Wainer, H. (1979). The Rasch model as additive conjoint measurement. Applied Psychological Measurement, 3(2), 237-255.
8. Newby, V. A., Conner, G. R., Grant, C. P., & Bunderson, C. V. (2009). The Rasch model and additive conjoint measurement. Journal of applied measurement.
9. Ofsted. Beyond 2012 - outstanding physical education for all. https://www.gov.uk/government/publications/beyond-2012-outstanding-physical-education-for-all2013
10. Eddy LH, Hill LJB, Mon-Williams M, Preston N, Daly-Smith A, Medd G et al. Fundamental Movement Skills and their Assessment in Primary Schools from the Perspective of Teachers. Research Square Pre-Print. doi:10.21203/rs.3.rs-32875/v1.
11. Routen AC, Johnston JP, Glazebrook C, Sherar LB. Teacher perceptions on the delivery and implementation of movement integration strategies: the CLASS PAL (physically active learning) Programme. International Journal of Educational Research. 2018;88:48-59.
12. Eddy LH, Bingham DD, Crossley KL, Shahid NF, Ellingham-Khan M, Otteslev A et al. The validity and reliability of observational assessment tools available to measure fundamental movement skills in school-age children: A systematic review. PLOS ONE. 2020;15(8):e0237919. doi:10.1371/journal.pone.0237919.
13. Bruininks R, Bruininks B. Bruininks-Oseretsky Test of Motor Proficiency (2nd Edition) Manual. Circle Pines, MN: AGS Publishing; 2005.
14. Zuvela F, Bozˇanić A, Miletić Đ. POLYGON–A new fundamental movement skills test for 8 year old children: construction and validation. Journal of sports science and medicine. 2011; 10:157–63. PMID: 24149309
15. Department of Education Western Australia. Fundamental movement skills: Book 2 –The tools for learning, teaching and assessment. 2013.
16. https://assets.publishing.service.gov.uk/government/uploads/system/uploads/attachment_data/file/239040/PRIMARY_national_curriculum_-_Physical_education.pdf
17. Klingberg B, Schranz N, Barnett LM, Booth V, Ferrar K. The feasibility of fundamental movement skill assessments for pre-school aged children. Journal of Sports Sciences. 2018;37:1-9
18. Eddy LH, Hill LJB, Mon-Williams M, Preston N, Daly-Smith A, Medd G et al. Fundamental Movement Skills and their Assessment in Primary Schools from the Perspective of Teachers. Research Square Pre-Print. doi:10.21203/rs.3.rs-32875/v1.
19. Klingberg B, Schranz N, Barnett LM, Booth V, Ferrar K. The feasibility of fundamental movement skill assessments for pre-school aged children. Journal of Sports Sciences. 2018;37:1-9
20. Eddy LH, Hill LJB, Mon-Williams M, Preston N, Daly-Smith A, Medd G et al. Fundamental Movement Skills and their Assessment in Primary Schools from the Perspective of Teachers. Research Square Pre-Print. doi:10.21203/rs.3.rs-32875/v1.
